# Complementary efficacy and molecular insights of *Polygonati rhizoma* polysaccharide for glycemic and lipid abnormalities in type 2 diabetes mellitus: a comprehensive review

**DOI:** 10.3389/fendo.2025.1606816

**Published:** 2025-09-10

**Authors:** Shichao Ma, Lu Xu, Yadi Hou, Wenjing Zhang, Yongxia Cui, Suiqing Chen

**Affiliations:** ^1^ College of Pharmacy, Henan University of Chinese Medicine, Zhengzhou, China; ^2^ Henan Provincial Key Laboratory of Chinese Medicine Resources and Chinese Medicine Chemistry, Henan University of Chinese Medicine, Zhengzhou, China; ^3^ Collaborative Innovation Center of Research and Development on the Whole Industry Chain of Yu-Yao, Henan University of Chinese Medicine, Zhengzhou, China

**Keywords:** *Polygonati rhizoma* polysaccharide, type 2 diabetes mellitus, glucose and lipid metabolism, molecular mechanisms, gut microbiota

## Abstract

**Background:**

T2DM is a global health challenge characterized by insulin resistance and impaired glucose metabolism. Natural products, such as polysaccharides from medicinal plants, are increasingly explored for their potential in managing T2DM with fewer side effects compared to conventional drugs.

**Aim:**

This review aims to comprehensively evaluate the effects of *Polygonati Rhizoma* Polysaccharide (PRP) on glucose and lipid metabolism in T2DM and elucidate its underlying molecular mechanisms.

**Introduction:**

In recent years, numerous scientific studies have demonstrated that *Polygonati Rhizoma* Polysaccharide (PRP) exerts beneficial effects on type 2 diabetes mellitus (T2DM), enhancing both glucose and lipid metabolism. This article provides a comprehensive review of the impact of PRP on T2DM influencing glucose and lipid metabolism and elucidates the underlying molecular mechanisms.

**Methods:**

A comprehensive literature search was conducted across electronic databases including PubMed, Web of Science, Wanfang Database, and China National Knowledge Infrastructure (CNKI). The search encompassed publications from June 2010 to June 2025, representing a 15-year period. Titles and abstracts were systematically screened for relevance, after which full-text articles meeting the inclusion criteria within this timeframe were selected for analysis.

**Discussion:**

Current evidence indicates that PRP can ameliorate T2DM alongside improving glucose and lipid profiles; this effect is predominantly linked to its modulation of the PI3K/AKT signaling pathway. Furthermore, its lipid-lowering properties are associated with regulation of gut microbiota, enhancement of intestinal barrier function, promotion of short-chain fatty acids (SCFAs) production, activation of G Protein-Coupled Receptors 41 and 43 (GPCR41 and 43), as well as inhibition of the TLR4/MyD88/NF-κB signaling cascade.

**Conclusion:**

*Polygonati Rhizoma* Polysaccharide can obviously improve the hyperglycemia and hyperlipidemia caused by T2DM, and has certain anti-inflammatory and antioxidant effects.

## 1 Introduction

Type 2 diabetes mellitus (T2DM) has emerged as a major global health challenge, consistently ranking among the top ten causes of disability, morbidity, and mortality across all age groups. According to the International Diabetes Federation (IDF) Diabetes Atlas (2025), diabetes affects 11.1% of adults aged 20–79 years, with over 40% of cases remaining undiagnosed. The IDF projects that the total number of affected individuals will surge to 783 million by 2045. This high prevalence is attributable to accelerating urbanization, population aging, reduced physical activity, and rising rates of overweight and obesity (1). T2DM is associated with several cardiovascular complications, including ischemic heart disease, heart failure, stroke, coronary artery disease, and peripheral artery disease. These conditions collectively account for at least 50% of deaths among diabetic patients (2). As no cure currently exists, investigating effective management and therapeutic strategies is imperative.

Diabetes is mainly categorized into type I and type II while, other types of diabetes such as gestational diabetes mellitus. T1DM is characterized by an absolute insulin deficiency caused by pancreatic cell destruction, while T2DM is mainly caused by insulin resistance (IR) and insufficient insulin secretion (3). It is widely acknowledged that diabetes is mainly caused by IR and β-cell dysfunction. However, in individuals at high risk for T2DM, IR emerges prior to the impairment of glucose homeostasis. Nevertheless, if β-cells can secrete enough insulin to counteract the effects of IR, glucose tolerance can be preserved (4).

A healthy dietary pattern, the reduction energy intake, and regular physical activity can partially suppress hyperglycemia and control T2DM (5). Nevertheless, due to the progress of T2DM and the difficulty of long-term lifestyle changes, most of the patients in clinic still need to resort to insulin and oral medications for treatment (6). Some medications can induce adverse reactions. For instance, metformin may cause dyspepsia and even lactic acidosis (LA) in some patients (7) and sulfonylureas can cause weight gain, fluid retention and hypoglycemia in some patients, thereby increasing the incidence of cardiovascular (CVD) disease (8).

In order to avoid the adverse reactions caused by these drugs, researchers have redirected their attention to natural products (NPs). Natural products, including herbal compounds and their extracts, have been employed to treat human diseases for thousands of years, and are being increasingly utilized in the treatment of type 2 diabetes (9). Bioactive metabolites isolated from natural medicinal plants are used in the treatment of diabetes by antioxidation, anti-inflammation, regulating glucose and lipid metabolism, islet cell function restoration, and ferroptosis regulating capabilities (10, 11). One such plant is *Polygonati Rhizoma*, commonly known as ‘Huang Jing’ in China, which has been used in traditional Chinese medicine for over a millennium to treat diabetes and related conditions. In China, “Huang jing” is one of the homologies of medicine and food. It and holds high edible and medicinal value (12, 13). The rhizomes of *Polygonatum sibiricum* Redouté. (PS), *Polygonatum cyrtonema* Hua. (PC) and *Polygonatum kingianum* Collet & Hemsl. (PK) are known as “Huang jing” (14). The pharmacological applications of *Polygonati Rhizoma* make it more and more popular in clinical practice for treating diseases, such as cancer (15–17), Alzheimer’s disease (18, 19), diabetes (20, 21), depression (22, 23). *Polygonati Rhizoma* contains many bioactive components, such as polysaccharides, saponins, flavonoids, phenols, alkaloids, anthraquinones, lignans, and a variety of beneficial amino acids (24). Polysaccharide is one of the most important components of *Polygonati Rhizoma* for its hypoglycemic effect, and the content of polysaccharide can be used as a marker for the quality indicator of *Polygonati Rhizoma* (14). The structural characteristics of polysaccharides—including molecular weight, monosaccharide composition, glycosidic linkages, and others—are fundamental determinants of their bioactivity. However, studies investigating the structure-activity relationships of *Polygonatum rhizome* polysaccharides (PRP) in the treatment of T2DM remain relatively limited, warranting further investigation. Current studies extensively investigate PRP’s ability to reduce blood glucose levels, enhance lipid metabolism, mitigate oxidative stress, and suppress inflammation, highlighting its considerable advantages in diabetes management.

While, several studies have explored the effects of PRP, a comprehensive review synthesizing its dual mechanisms in regulating glycemic and lipid abnormalities in T2DM is currently lacking. This review evaluates the therapeutic roles of PRP derived from three *Polygonatum* species in managing T2DM and summarizes their underlying molecular mechanisms. Thus, this review is novel in its integrative analysis of PRP’s dual effects on glucose and lipid metabolism in T2DM, consolidating recent studies not previously synthesized. This review comprehensively examines PRP’s effects on glucose/lipid metabolism and proposes a novel dual-intervention mechanism for T2DM glucose-lipid abnormalities. It further details key molecular pathways – including PI3K/AKT signaling and gut microbiota regulation – laying the groundwork for future therapeutic development.

## 2 Search methodology

### 2.1 Study design and search criteria

This review includes the current research findings and presents an overarching panorama of the impacts of three different flavin-derived polysaccharides on dysglycemia and dyslipidemia rat models of T2DM. We searched PubMed, Web of Science, Wanfang databases and China National Knowledge Infrastructure (CNKI) from their inception to June 2025. The language of the retrieved literature was confined to English and Chinese. The following terms were used to scour for articles by title, summary, or keywords: “Polygonatum”, “Polygonatum sibiricum”, “Polygonatum sibiricum polysaccharide”, “Polygonatum kingianum”, “Polygonatum kingianum polysaccharide”, “Polygonatum cyrtonema”, “Polygonatum cyrtonema polysaccharide”, “Polygonatum rhizoma”, “Diabetes Mellitus, Type2”, “Type 2 diabetes mellitus”, “Type 2 diabetes”, “T2DM” and “Noninsulin-Dependent Diabetes Mellitus”. Analogous search combinations were used for particular databases.

### 2.2 Data charting process

The screening of articles mainly includes two steps. Firstly, a preliminary screening is executed in accordance with the abstract and title, and then the articles screened in are evaluated in full text to ascertain they meet the inclusion criteria. After screening, the clinical validation and mechanism analysis were analyzed separately. Subsequently, the following details were extracted from the selected studies: (1) the name of the first author and year of publication; (2) characteristics of the study animals (species, sample size, age, sex and weight); (3) information of the treatment and control group (countermeasures, duration of treatment, and dosage); (4) outcome indicators. In addition to glycemia and blood lipid, other relevant parameters were also taken into consideration. The relevant parameters analyzed in this study include: hemoglobin A1c(HbA1c), fasting/postprandial blood glucose, low-density lipoprotein cholesterol (LDL-C), high-density lipoprotein cholesterol (HDL-C), triglyceride, and so forth.

### 2.3 Eligibility criteria

Studies were considered eligible if they met the following inclusion criteria: (i) a study model of T2DM as the primary disorder; (ii) availability of full-text and articles published in English or Chinese; (iii) Polygonatum rhizoma extract or powder. The exclusion criteria included: (i) Type 1 diabetes mellitus; (ii) abstracts or conference paper comments, (iii) absence of peer-reviewed articles, proceedings, and letters/comments.

## 3 Results

### 3.1 Literature search and study flowchart

**Figure 1:**
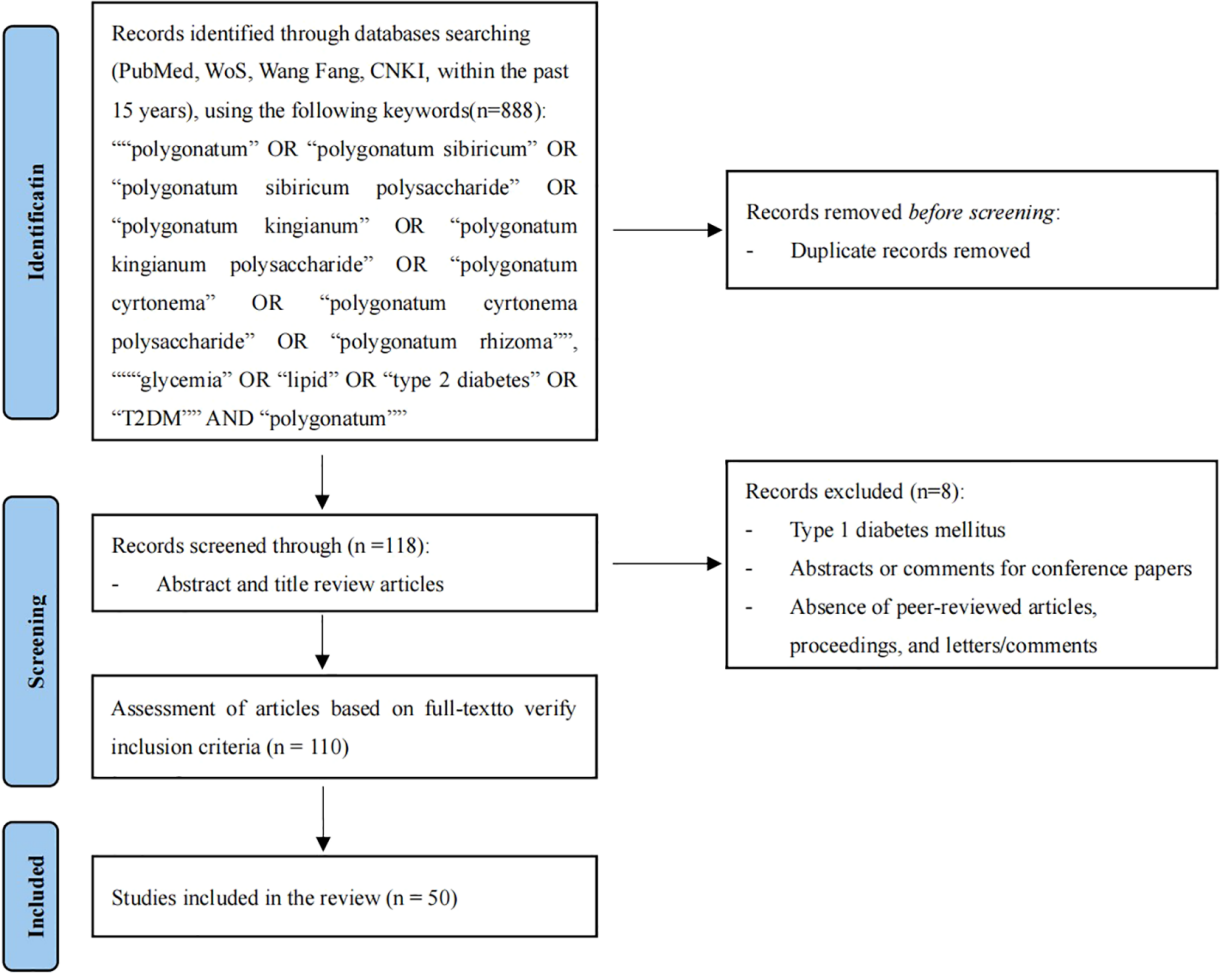
Flow diagram depicting the study selection process.

Initially, a total of 888 articles on the reduction of blood glucose and lipids by PRP were retrieved from the database. Among them, 453 papers were retrieved from PubMed, 106 from WoS, 103 from CNKI, and 226 from Wangfang. After meticulous review of the titles, 118 articles were selected, among which 80 focused on the effects of PRP on blood glucose and 38 on its effects on lipids. Following systematic screening of titles and abstracts, this review incorporated 50 articles investigating the mechanisms by which PRP ameliorate type 2 diabetes mellitus (T2DM). Among these, 23 studies focused exclusively on PRP’s glucose-lowering effects, with several concurrently documenting additional benefits such as renal protection and antioxidant activity. Twelve studies specifically explored PRP’s lipid-regulating mechanisms, some revealing ancillary improvements in atherosclerosis and gut microbiota modulation. Notably, 15 studies definitively demonstrated PRP’s dual efficacy in simultaneously ameliorating hyperglycemia and dyslipidemia. Collectively, this evidence highlights PRP’s multi-target potential in countering T2DM through interconnected biological pathways (**Figure 1**), with the complete literature screening list provided in **Supplementary Materials** (**Supplementary Table S1**).

### 3.2 Hypoglycemic efficacy of *Polygonati rhizoma* polysaccharide in T2DM rodent models

This review analyzed 23 studies that directly investigated the glucose-lowering effects of three distinct *Polygonatum* polysaccharides in T2DM rats (with an additional 15 studies addressing both glucose and lipid regulation). The findings demonstrate that PRP significantly reduced blood glucose concentrations in T2DM animal models. Specifically, 30 publications reported substantial reductions in fasting blood glucose (FBG) or HbA1c levels across diverse T2DM induction models. Improved insulin sensitivity was documented in 11 studies, while enhanced glucose tolerance was observed in 8 studies. Critically, across all glucose-lowering investigations, T2DM models treated with PRP consistently exhibited lower blood glucose levels compared to untreated controls.

In a streptozotocin (STZ)-induced T2DM mouse model, *P. sibiricum* polysaccharide (PSP) was administered via gavage at doses of 200, 400, and 800 mg/kg/day. The concentrations of FBG and HbA1c exhibited a progressive decline corresponding to the escalating PSP dosage, indicating a dose-dependent response. Notably, significant variations in FBG and HbA1c levels were discerned across the experimental groups (P<0.05) (25). Polysaccharide fractions (PSF) and total polysaccharides (PS) from *P. kingianum* were orally administered to obese Sprague-Dawley rats at doses of 120/240/480 mg/kg/day for a duration of 14 weeks. Following treatment, a marked reduction in FBG levels was observed in both PSF and PS groups, with a statistical significance of (P < 0.001) (26).

Li’s study demonstrated that *P. sibiricum* polysaccharide primarily participates in hepatic metabolism, and its mechanism of action in the liver primarily linked to glucose metabolism (27). Rats with blood glucose levels exceeding 16.7 mmol/L experienced a noteworthy reduction in FBG following continuous treatment with 200 and 400 mg/kg/day of *P. sibiricum* polysaccharide over a 4-week period (28). *P. kingianum* aqueous extract can significantly reduce blood glucose and alleviate skin damage caused by advanced glycation end products deposition in rats with diabetic skin lesions (29). After intragastric administration of 0.5, 1 and 2g/kg water extract of *P. sibiricum* for 6 weeks, the levels of FBG and HbA1c in mice were significantly decreased (30). These results robustly support the significant beneficial hypoglycemic effects of PRP in the treatment of T2DM.

The results consistently indicate a significant reduction in FBG levels following the administration of PRP. This implies its potential application as a therapeutic agent for blood glucose control. Furthermore, an investigation into the underlying mechanism revealed that PRP has the potential to enhance insulin sensitivity by activating the PI3K/AKT pathway, thereby facilitating improved cellular glucose uptake (31–34).

### 3.3 Anti-atherosclerotic and lipid-lowering efficacy of *Polygonatum rhizoma* polysaccharides in T2DM rodent models

This review analyzed 12 studies directly investigating the efficacy of three distinct PRPs in improving lipid metabolism within T2DM rat models, along with 15 additional studies examining both glycemic and lipid regulation. The findings collectively demonstrate that PRP treatment significantly reduced serum lipid levels in T2DM animal models. Specifically, 21 studies reported decreased total cholesterol (TC), triglycerides (TG), and LDL levels, or significantly elevated HDL levels in PRP-treated T2DM animals. The underlying mechanisms primarily involve: (1) inhibiting key lipid synthesis factors (e.g., SREBP-1c; supported by 4 studies), (2) promoting fatty acid oxidation (e.g., via activation of the PPARα/CPT-1 pathway; supported by 3 studies), and (3) modulating the gut microbiota (e.g., increasing *Lactobacillus* abundance and the Firmicutes/Bacteroidetes ratio; supported by 13 studies). These beneficial effects demonstrated consistent efficacy across diverse models, including high-fat diet and atherosclerosis, and exhibited cross-species reproducibility.

Guo’s study demonstrated the beneficial influence of *P. cyrtonema* polysaccharide (PCP) on dyslipidemia and atherosclerosis induced by a high-fat diet (HFD) in both female and male rats, with a more pronounced effect observed in males (35). Similarly, Yang’s investigation revealed that *P. sibiricum* polysaccharides, at varying concentrations, effectively mitigated blood lipids and exhibited anti-atherosclerotic properties in obese rabbits. These anti-atherosclerotic effects were attributed to the direct reduction of blood lipids and the protection of endothelial cells from apoptosis and necrosis (36). In Zeng’s research, gavage treatment with *P. sibiricum* polysaccharides (PSPs) at different concentrations (200/400/800 mg/kg) over six weeks resulted in reduced body weight, serum TC, TG, and LDL-C levels in HFD-induced rats (37). Additionally, both the ethanol extract (ID1216) and the water extract (PSAE) of *P. sibiricum* significantly attenuated weight gain, serum TC, and triglyceride levels, concurrently improving hepatic insulin resistance (IR) in obese mice (30, 38).

Despite the optimistic findings, it is essential to recognize the need for rigorous clinical trials to validate these outcomes in human. Moreover, exploring potential side effects and the optimal dosage of PRP becomes crucial for its safety and effection. In conclusion, the current evidence suggests a promising lipid profile effect of PRP, marking it a noteworthy subject for future research in the realm of metabolic disorders. **Table 1** presents 15 studies that accurately investigate the effects of three different sources of PRP on improving blood glucose and blood lipids in animals with T2DM.

**Table 1:** Effects of *Polygonati rhizoma* polysaccharide on blood glucose and blood lipids in T2DM and impaired glucose tolerance animals.

No.	References	Species	Component name	Species (sex, n=, treatment/ blank control group, age, weight)	Established model	Modeling standard	Experimental group (approach, daily, dosage, duration)	Blank control group	Positive control group	Index
1	Gu et al. (1)	*P.kingianum*	*P.kingianum* polysaccharide fractions (PSF), *P.kingianum* total polysaccharides (PS)	SD rats (NM,5/5, NM,200±20g)	HFD	NM	Intragastric,120/240 and 480mg/kg/day,14 weeks	Equal volume of NS	1.8mg/kg/day SIM	Comparison between experimental and model groups: PSF.H, PS.L and PS.M:BWG↓(P<0.05);PSF and PF: PPARγ↑(P<0.001);PS:TC,TG↓(P<0.05);PSF:LDL-C↓(P<0.01);PSF and PS:HDL-C↑(P<0.001),IL-10↑(P < 0.01),TLR4↓(P<0.001),IL-1β↓(P<0.01)
2	Wang et al. (2)	*P. sibiricum*	*P. sibiricum* polysaccharide (PSP)	SD rats (Male,96/24,7 weeks,200±20g)	SIJ+STZ (60mg/kg with 2% sodium citrate buffer solution, pH 4.44)	FBG>13.9mmol/L	Intragastric,200/400 and 800mg/kg/day,12 weeks	Equal volume of NS	NM	Comparison between experimental and model groups:PSP-L,PSP-M,PSP-H:FBG,HbA1c↓(P<0.05),Bax,EGF,p38,TGF-β and VEGF↓(P < 0.01); Bcl-2↑
3	Li et al. (3)	*P.kingianum*	A novel *P.kingianum* polysaccharide (PKPs-1)	ICR mice (Male,10/10,6 weeks,20±2g)	HFD+STZ (120mg/kg with 0.1 M sodium citrate buffer, pH 4.5)	BG>11mmol/L	Intragastric,1190mg/kg/day,15 days	Equal volume of NS	150 mg/kg/day MET	Comparison between experimental and model groups:PKPs-1:FBG↓(P <0.001),TC,TG,LDL-C↓(P<0.01);HDL-C,IRS1/PI3K/Akt↑(P<0.01)
4	Wang et al. (4)	*P.sibiricum*	*P.sibiricum* polysaccharide (PSP)	SD rats (Male,56/16,2 months,200±20g)	Single tail vein injection STZ (60mg/kg with citrate buffer)	BG >13.9 mmol/L at 3 and 7 days after STZ injection	Intragastric,200/400 and 800mg/kg/day,12 weeks	NS(1ml/100g/day)	NM	Comparison between experimental and model groups:PSP-L,PSP-M,PSP-H:FBG,HbA1c↓(P<0.01);INS↑(P<0.05),C-peptide↑(P < 0.05 or P < 0.01)
5	Zeng et al. (5)	*P.sibiricum*	*P.sibiricum* polysaccharide (PSP)	SD rats (Male,32/8, NM,145-160g)	HFD	NM	Intragastric,200/400 and 800mg/kg/day,6 weeks	Equal volume of distilled water	NM	Comparison between experimental and HFD groups : PSP: ALT,AST↓(P<0.01);PSP400 and PSP800:BW↓(P<0.05 and P<0.01),TC,TG↓(P<0.01);PSP800: LDL-C↓ (P < 0.01), HDL-C↑ (P < 0.01); PSP200 and PSP400: HDL-C↑ (P < 0.05)
6	Guo et al. (6)	*P.cyrtonema*	*P.cyrtonema* polysaccharide (PCP)	LDLr mice (Male and female,45/15,8 weeks,20±2g)	HFD	NM	Intragastric,65 and 260 mg/kg/day,16 weeks	Equal volume of NS	ACT,2 mg/ (kg ·day)	Comparison between experimental and HFD groups : PCP:BWG↓ (P < 0.05);PCP260(female and male):TC, LDL-C and AI↓(P < 0.01),TG↓(P<0.05 and P<0.01),HDL-C↑(P<0.05 and P<0.01);PCP65(female and male):TC↓(P<0.05),LDL-C↓(P<0.05 and P<0.01),TG↓(No effect and P<0.05),HDL-C↑(P<0.05 and P<0.01)
7	Jia et al. (7)	*P.sibiricum*	*P.sibiricum* polysaccharide (PSP)	BALB/c mice (Male,10/10,2 months,18±2g)	HFD	BG>10mmol/L	Intragastric,500mg/kg/day,8 weeks	Equal volume of DW	NM	Compared with the HFD group: BG ↓at 15, 30, 60 and 120 min(P<0.05),FBG,INS↓(P<0.05);IRS-2↑(P<0.05);No significant effect on BW
8	Zeng et al. (8)	*P.sibiricum*	*P.sibiricum* polysaccharide (PSP)	KM mice (NM,40/10, NM,20±2g)	Continuous STZ intraperitoneal injections for 5 days (50 mg/kg)	NM	Intragastric,100/200 and 400mg/kg/day,28 days	Equal volume of NS	250 mg/kg/day MET	Compared with the model group:TC↓(P<0.05),TG↓(P<0.01),INS↑,HG↑(P<0.01),SOD↑(P<0.05),GSH-Px↑(P<0.01),MDA↓(P < 0. 05, P < 0. 01),the mRNA expression levels of Akt, PI3K, IRS1, PDK1, GLUT2, PIP5K, and GSY↑(P < 0.05, P < 0.01);PSP200 and PSP400: GSK-3β↑ (P < 0.05, P < 0.01)
9	Zhang et al. (9)	*P.kingianum*	*P.kingianum* total polysaccharide(PPS) and *P.kingianum* homogeneous polysaccharide (PPS1)	ICR mice (Male,45/15, NM,18-20g)	HFD+Single tail vein injection STZ (120mg/kg)	BG>11.1mmol/L	Intragastric,1.19 and 1.19g/kg/day,15 days	NS(1ml/100g/day)	150 mg/kg/day MET	After 5, 10 and 15 days of continuous administration,compared with the model group: PPS:FBG↓((P < 0.01,P < 0.01,P < 0.01);PPS1:FBG↓(P < 0.01, P < 0.05, P < 0.01);Compared with the model group: PPS:TC↓(P< 0.01),TG↓(P< 0.01),TC/HDL↓(P< 0.01),LDL↓(P< 0.01),HDL↑(P< 0.05),HG,MG↑(P<0.05);PPS1:TC↓ (P< 0.01), TG↓ (P< 0.05), LDL↓ (P< 0.01)
10	Xu (10)	*P.cyrtonema*	*P.cyrtonema* polysaccharide (PCP)	C57BL/6 mice (Male,30/10, NM,18±2g)	HFD	NM	Intragastric,250/500 and 1000mg/kg/day,12 weeks	Equal volume of DW	NM	Comparison between experimental and model groups:PCP1000:BG,INS↓(P<0.05),BWG,Lee's index,and fat index↓, TC,TG,LDL-C↓(P<0.05);PCP500:TC,Lee's index and fat index↓(P<0.05);PCP250: fat index↓ (0.05); No effect in ΔHDL-C
11	Bao (11)	*P.sibiricum*	*P.sibiricum* polysaccharide (FPSP)	KM mice (NM,40/10, NM,20±2g)	HFD	NM	Intragastric,100/200 and 400mg/kg/day,8 weeks	Equal volume of NS	1.8mg/kg/day SIM	Comparison between intervention and control groups:GZ and GG:BW,fat index↓(P<0.05);GD,GZ and GG:liver index↓(P<0.05);GG:TC,TG,LDL-C↓(P<0.05),HDL-C↑(P<0.05)

NM, not mentioned; NS, normal saline; SIM, Simvastatin; BW, body weight; BWG, body weight gains; PPARγ, peroxisome proliferator-activated receptors-g; MOD, high-fat diet lipid metabolism disorder model group;IL-10, 1β interleukin-10, 1 beta; SIJ, single intraperitoneal injection; Bcl-2, B-cell lymphoma-2; Bax, BCL2-Associated X; EGF, epidermal growth factor; p38, p38 MAPK; TGF-β,transforming growth factor-β; VEGF, vascular endothelial growth factor; BG, blood glucose; MET, metformin; DM, diabetes mellitus; INS, insulin; ALT, alanine aminotransferase; AST, aspartate aminotransferase; ND, normal diet group; AI, atherosclerotic index; IRS-2, insulin receptor substrate 2; DW, distilled water; SOD, superoxide dismutase; GSH-Px , glutathione peroxidase; HG, hepatic glycogen; MG, muscle glycogen; MDA, malondialdehyde; Lee's index,(weight *1000)^(1/3)/ length (cm); GD, low dose FPSP; GZ, middle dose FPSP; GG, high dose FPSP; GM, Model group; FFAs, free fatty acids; GSP, glycated serum proteins; HOMA-IR (homeostasismodel assessment-insulin resistance)=(FBG×FINS)/22.5; FINS, fasting blood insulin; Cr, kidney function-related markers creatinine; BUN, blood urea nitrogen.

### 3.4 *Polygonati rhizoma* polysaccharide ameliorates T2DM-associated inflammation and oxidative stress

Studies indicate that PRP not only reduces blood glucose and lipids but also mitigates inflammation and oxidative stress induced by T2DM. Twelve publications demonstrate that PRP effectively lowers pro-inflammatory cytokine levels (e.g., TNF-α, IL-6, IL-1β) or suppresses inflammatory responses. Furthermore, several studies (25, 39–41) reveal its protective effects against complications such as diabetic nephropathy, myocardial fibrosis, and retinopathy. Thirteen additional publications confirm that PRP enhances antioxidant enzyme activity (including SOD, GSH-Px, and CAT) or reduces oxidative damage markers (e.g., MDA, H_2_O_2_, ROS), thereby improving systemic antioxidant capacity and alleviating oxidative stress.

Research has established the inhibitory effects of *P. sibiricum* polysaccharide (PSP) on interleukin (IL)-1β, IL-6, and tumor necrosis factor (TNF)-α expression across multiple models: LPS-stimulated RAW264.7 macrophages, ovariectomized rats, and acute heart failure models, collectively demonstrating PSP’s anti-inflammatory potential (42–44). Liu’s study showed that *P. sibiricum* polysaccharide may have lipid-lowering and anti-inflammatory effects by activating the AMPK pathway (45). *P. sibiricum* polysaccharide mitigates the inhibitory effects of palmitic acid (PA) on skeletal muscle cell survival, inflammation, and glucose uptake. This improvement is attributed to the inhibition of miR-340-3p expression, a versatile miRNA involved in cell survival, apoptosis, and differentiation (46). Additionally, *P. cytonema* polysaccharide significantly reduces pro-inflammatory cytokines (TNF-α, IL-1β, and IL-6) while enhancing the expression of key antioxidant genes, including superoxide dismutase 1 (SOD1), glutathione peroxidase 2 (GPX2), and nuclear factor erythroid 2-related factor 2 (Nrf2) (47). The *in vitro* anti-inflammatory activity of PKP2-1, a novel *P. kingianum* polysaccharide, on MH7A cells showed that PKP2–1 reduced the expression of IL-11β and IL-6, increased the expression of IL-10, and induced apoptosis of synovial fibroblasts (48).

Research indicates a crucial involvement of oxidative stress in hyperglucose-induced tissue damage and early events associated with the onset of T2DM, potentially contributing to the destruction of β-cells in individuals with T2DM (49). Research indicates that PRP possesses the capability to neutralize free radicals, thereby reducing oxidative damage. Its robust anti-oxidative effect is primarily attributed to the modulation of the Nrf2/HO-1 antioxidant signaling pathway (20, 50–52). Steaming process markedly enhances *P. sibiricum*’s ability to scavenge free radicals. Furthermore, research suggests that the fourth steaming cycle effectively improves the antioxidant activity of *P. sibiricum* polysaccharide (53). An alternative investigation demonstrated that microwave-assisted degradation effectively enhances the antioxidant activity of *P. sibiricum* polysaccharide through the reduction of its molecular weight (54). *P. cytonema* polysaccharides (PCHPs) not only have antioxidant activity, but also have a certain degree of antibacterial activity (55). Zhao’s research demonstrates the remarkable antioxidant capacity of the polysaccharide PCP-F1 extracted from *P. cytonema* (56).

Collectively, these findings demonstrate PRP’s significant efficacy in ameliorating diabetes-related inflammation and oxidative damage. PRP effectively mitigate oxidative damage by directly activating antioxidant pathways (such as Nrf2/HO-1) and indirectly modulating the metabolic-inflammatory network. PRP demonstrates significant antioxidant efficacy across various disease models, including diabetes, obesity, nephropathy, retinopathy, and atherosclerosis.

### 3.5 The clinical role of *Polygonatum rhizoma* as a component in Chinese medicine formulas for T2DM management

This article has compiled clinical studies on *Polygonati Rhizoma* as a component of traditional Chinese medicine formulas for treating T2DM over the past 15 years. A total of 14 articles has been included. Among them, Zhang and Li (2007)’s (57) study on Polygonatum (Huangjing) Decoction, which used *Polygonatum* alone for the treatment of T2DM and achieved remarkable results, was also included. Classify the *Polygonati Rhizoma* (also known as “Monarch-Minister-Assistant-Envoy”) in different traditional Chinese medicine compound prescriptions based on the *Jun-Chen-Zuo-Shi* theory. Among them, there are 4 articles referring to *Polygonati Rhizoma* as “Monarch”, 7 articles as “Minister”, and 3 articles as “Assistant” (**Table 2**).

**Table 2:** *Polygonati rhizoma*, as a component of traditional Chinese medicine formulas, has been used in some clinical studies for the treatment of T2DM.

No.	References	Formula name	Role/dose of polygonatum (Monarch/minister /assistant/envoy)	Polygonatum weight proportion	Diagnostic criteria (T2DM)	Study-design (n=Sample, per group)	Intervention	Control	Primary outcomes
1	Gong et al. (1)	Compound *Polygonatum sibiricum* prescription	Monarch 20 g/day	44.4% (20g/45g total formula)	1.FPG ≥7.0 mmol/L 2. 2hPG during OGTT ≥11.1 mmol/L 3.Random plasma glucose ≥11.1 mmol/L (meeting any one criterion)	RCT (n=64,32/32)	Metformin (850mg/day) + Lifestyle + Polygonatum Formula (1 dose/day) × 12 weeks	Metformin (850mg/day) + Lifestyle	↓ FBG (P<0.05 vs. control); ↓ HbA1c (P<0.05 vs. control); ↓HOMA-IR (P<0.05 vs. control); ↓ TC; TG (P<0.05 vs. control); Total Efficacy Rate 87.5% vs. 59.4% (P<0.05 vs. control)
2	Cai et al. (2)	Huangjing Qianshi Decoction	Monarch 15 g/day	11.9% (15g/126g total formula)	Prediabetes (IGR): FPG 6.1–7.0 mmol/L or 2hPG 7.8–11.1 mmol/L	RCT (n=80,40/40)	Huangjing Qianshi Decoction (1 dose/day) + Lifestyle intervention (diet + exercise) × 24 weeks	Lifestyle intervention (diet + exercise) only	↓ FPG (P<0.05 vs. control); ↓ 2hPG (P<0.05 vs. control); TCM Symptom Score: ↓↓ Significant improvement (both groups, P<0.05), but no intergroup difference (P >0.05)
3	Zhang & Li (3)	Polygonatum (Huangjing) Decoction	Monarch 50g/day	100% of formula	1.FPG ≥7.0 mmol/L 2. 2hPG during OGTT ≥11.1 mmol/L 3.Random plasma glucose ≥11.1 mmol/L (meeting any one criterion)	RCT (n=94,48/46)	Huangjing decoction: 50g/day Duration: 1 months	Metformin: 50–100mg BID	↓ FPG (P<0.05 vs. control); ↓ 2hPG (P<0.05 vs. control); Efficacy Rate:81.25% (vs. 56.52% in control, P<0.05)
4	Bai et al. (4)	Sanhuang Tangmin Decoction	Monarch 15g/day	17.86% (15g/84g total formula)	Western: 1.FPG ≥7.0 mmol/L 2. 2hPG during OGTT ≥11.1 mmol/L 3.Random plasma glucose ≥11.1 mmol/L (meeting any one criterion) BMI≥24 TCM: Qi-Yin deficiency + blood stasis/dampness	RCT (n=100,60/40)	Sanhuang Tangmin Decoction: 300 mL/day (150 mL bid) + Metformin: 0.5g BID (1g/day) × 8 weeks	Metformin: 0.5g BID (1g/day)	↓ FPG (P<0.05 vs. control); ↓ 2hPG (P<0.05 vs. control); Efficacy Rate: 80.0% (vs. 62.5%, P<0.05)
5	Yan & Li (5)	Xiaoke No.1 Decoction	Minister 15 g/day	6.91% (15g/217g total formula)	Western: Lab-confirmed (FPG=13.2 mmol/L) TCM: Lung-Kidney Yin Deficiency	Case report (n=1)	Xiaoke No.1 + Insulin/Metformin/Acarbose × 6 weeks	None	FPG: 13.2→5.1 mmol/L
6	Huang & Lu (6)	Xiaoke No.2 Decoction	Minister 15 g/day	9.1% (15g/165g total formula)	Western: 1. Random plasma glucose ≥11.1 mmol/L 2. FPG ≥7.0 mmol/L 3. 2hPG during OGTT ≥11.1 mmol/L 4. HbA1c ≥6.5% (meeting any one criterion) TCM: Qi-Yin Deficiency	RCT (n=60,30/30)	Xiaoke No.2 + Metformin XR (1g/day) Duration: 3 months	Metformin XR (1g/day)	↓ FPG: -3.58 mmol/L (P<0.05 vs. control); ↓ ΔHbA1c: -1.80% (P<0.05 vs. control) Total efficacy: 90% (P<0.05 vs. control)
7	Feng (7)	Shenmai Yiqi Yangyin Decoction	Minister 15 g/day of Zhi Huangjing (processed Polygonatum)	7.43% (15g/202g total formula)	1. Polydipsia, polyphagia, polyuria, unexplained weight loss 2. Random plasma glucose ≥11.1 mmol/L 3. FPG ≥7.0 mmol/L 4. 2hPG during OGTT ≥11.1 mmol/L 5. HbA1c ≥6.5% (meeting any one criterion)	RCT (n=160,80/80)	Shenmai Yiqi Yangyin (1 dose/day, 100 mL BID) + Gliclazide MR (30→60 mg/day after 30 days) × 8 weeks	Gliclazide MR (30→60 mg/day after 30 days) × 8 weeks	↓ FPG, 2hPG, HbA1c (P<0.01 vs. control); ↓ HOMA-IR; ↑ HOMA-β (P<0.01 vs. control); ↑ TRF/Alb/PA/Hb (P<0.01 vs. control); ↑ ADPN/NO/FMD; ↓ ET (P<0.01 vs. control) ↑ IL-10; ↓ IL-6/TNF-α (P<0.01 vs. control)
8	Liu & Du (8)	Decoction of Nourishing Kidney and Tonifying Spleen	Minister 12g/day	6.03% (12g/199g total formula)	Western: FPG≥7.0 mmol/L or 2hPG≥11.1 mmol/L (meeting any one criterion) TCM: Spleen-kidney deficiency	Multicenter RCT (n=73,37/36), parallel-group	Decoction of Nourishing Kidney and Tonifying Spleen: 200 mL/day + Metformin: 500 mg TID (1.5 g/day) × 12 weeks	Metformin only: 500 mg TID (1.5 g/day)	↓HbA1c:(P<0.01 vs. control); Sperm Quality: ↑Forward motility (P<0.01 vs. control) ↑Normal morphology (P<0.01 vs. control)
9	Hu et al. (9)	Yiyuan Qinggan Jianyun Decoction	Minister 10g/day	5.75% (15g/174g total formula)	Western: 1.FPG ≥7.0 mmol/L 2. 2hPG during OGTT ≥11.1 mmol/L 3.Random plasma glucose ≥11.1 mmol/L (meeting any one criterion) CHO>6.2 mmol/L, TG>2.0 mmol/L, LDL-C>3.1 mmol/L, HDL-C<1.04 mmol/L	Controlled clinical trial ( n=240,120/120)	Yiyuan Qinggan Jianyun Decoction: 200 mL/day + hypoglycemic agents (unspecified)× 2 months	Fenofibrate (200 mg/day) + hypoglycemic agents (same as intervention group)	↓TG (P<0.05 vs. control); ↓LDL-C (P<0.05 vs. control); ↓FBG (P<0.05 vs. control); Total efficacy: 83.3% vs. 58.3% in control (P<0.05);
10	Ji et al. (10)	Bushen Huoxue Formula	Minister 12 g/day	7.41% (12g/162g total formula)	Western: 1.FPG ≥7.0 mmol/L 2. 2hPG during OGTT ≥11.1 mmol/L 3.Random plasma glucose ≥11.1 mmol/L (meeting any one criterion) TCM Pattern:Qi-Yin Deficiency Qi-stagnation and blood stasis	RCT (n=60,30/30)	Insulin + Bushen Huoxue Formula (6g TID) × 12 weeks	Insulin + Qianggu Capsule Dose: 0.25g TID	↓ FBG, ↓ 2hPG (P<0.05 vs. control); ↓ HbA1c (P<0.01 vs. control); ↓ TC (P<0.01 vs. control); ↓ TG (P<0.05 vs. control)
11	Li et al (11)	Xiaokefang	Minister 12g/day	6.94% (12g/173g total formula)	1. FBG≥7.0mmol/L 2. 2hPG ≥11.1 mmol/L Yin deficiency with heat syndrome (TCM)	RCT (n=90,45/45)	Xiaokefang Decoction: Daily dose (200-300 mL) + Insulin: Novolin N (start: 0.1 IU/kg, titrated to FBG≤6.5 mmol/L) × 8 weeks	Insulin only: Novolin N (start: 0.1 IU/kg, titrated to FBG≤6.5 mmol/L)	↓2hPG; ↓ HbA1c (P<0.05 vs. control); ↓32% insulin dose (Week 8, P<0.01) ↓87% hypoglycemia (P<0.01 vs. control) ↓Yin deficiency-heat score (P<0.01 vs. control)
12	Pan et al. (12)	Bushen Jianpi Recipe	Assistant 12 g/day	7.41% (12g/162g total formula)	Western: 1. Random plasma glucose ≥11.1 mmol/L 2. FPG ≥7.0 mmol/L 3. 2hPG during OGTT ≥11.1 mmol/L 4. HbA1c ≥6.5% (meeting any one criterion) TCM Pattern: Spleen-Kidney Deficiency	Prospective RCT (n=90,30/30/30)	TCM Group: Metformin 1g/day + Bushen Jianpi Recipe (400 mL/day) × 8 weeks Combination Group: Metformin 1g/day + Atorvastatin (20 mg/day) + Bushen Jianpi Recipe× 8 weeks	Metformin (1g/day) + Atorvastatin (20 mg/day) × 8 weeks	↓ FPG (P<0.01 vs. control); ↓ HbA1c (P<0.01 vs. control); ↓ LDL-C (P<0.05 vs. control); ↑ ADP (P<0.05 vs. control); TCM Efficacy: ↑ Symptom improvement (90.0% vs. 66.7%; P<0.05 vs. control)
13	Xiao et al. (13)	Huoxue Jiangtang Yin	Assistant Not specified	Unknown	Western: FPG≥7.0 mmol/L or 2hPG≥11.1 mmol/L (meeting any one criterion) TCM: Qi deficiency with blood stasis	RCT (n=84,42/42)	Huoxue Jiangtang Decoction: 240 mL/day (120 mL bid) + Metformin: 500 mg TID (1.5 g/day) × 20 weeks	Metformin only: 500 mg TID (1.5 g/day)	↓FPG; ↓HbA1c; ↓TC; ↓LDL-C (P<0.01 vs. control); ↓HOMA-IR (P<0.05 vs. control); ↓TNF-α (P<0.05 vs. control); ↑SFRP5(P<0.05 vs. control)
14	Xu & Liang (14)	Yiqi Tongluo Decoction	Assistant 30g/day	18.99% (30g/158g total formula)	1.FPG ≥7.0 mmol/L 2. 2hPG during OGTT ≥11.1 mmol/L 3.Random plasma glucose ≥11.1 mmol/L (meeting any one criterion)	RCT (n=71,37/36)	Conventional therapy + Yiqi Tongluo Decoction (1 dose/day) × 6 weeks	Conventional therapy + Atorvastatin Calcium (20mg/day)	↓ FBG (P<0.05 vs. control); ↓ TC (P<0.05 vs. control); ↓ TG (P<0.05 vs. control)

FPG, Fasting Plasma Glucose; 2hPG, 2-hour plasma glucose; TRF, Transferrin; Alb, Albumin; PA, Prealbumin; Hb, Hemoglobin; ADPN, Adiponectin; NO, Nitric Oxide; FMD, Flow-Mediated Dilation; SFRP5, Secreted Frizzled Related Protein 5; BID, twice daily; TID, three times daily; HOMA-IR, Homeostatic Model Assessment for Insulin Resistance.

Based on a comprehensive analysis of 14 clinical studies, *Polygonati Rhizoma* — serving as a core component in traditional Chinese medicine formulations — significantly reduces blood glucose and lipids in diabetic patients through indirect mechanisms. These include regulating glycolipid metabolism, suppressing inflammation (↓TNF-α/IL-6), improving insulin sensitivity (↓HOMA-IR), and enhancing vascular function (↑NO/FMD), while concurrently alleviating complications such as retinopathy, nephropathy, and sexual dysfunction.

## 4 Underlying mechanism of action of *Polygonati rhizoma* poly-saccharide in T2DM

The majority of investigations into the treatment of T2DM using PRP have centered on preclinical studies. Numerous animal experiments have demonstrated the potential of PRP in ameliorating both blood glucose and lipid profiles (28, 58, 59). Although multiple studies have detailed the advantageous metabolic effects of PRP on T2DM, the precise mechanism of action remains incompletely elucidated. This review’s research on mechanisms is entirely based on 50 screened literatures. Isolated suggestions, extracted from the literature, include the following:

### 4.1 Molecular mechanisms of glucose regulation induced by *Polygonati rhizoma* polysaccharide

In the T2DM rat group treated with *P. sibiricum* polysaccharide (PSP), intragastric administration of 500mg/kg once daily for 8 weeks resulted in a significant reduction in both FBG and insulin levels when compared to the HFD group (P<0.05). Simultaneously, there was a noteworthy increase in the expression of the insulin receptor IRS-2 (P < 0.05) (60). *P. sibiricum* polysaccharide (PSP) at doses of 200/400/800mg/kg was administered to STZ-induced diabetic rats. Over a 12-week period, this treatment led to a reduction of FBG and HbA1c levels, accompanied by improvements in symptoms of diabetes, such as polydipsia, polyphagia, and polyuria. Notably, PSP also demonstrated efficacy in alleviating diabetic retinopathy in rats (61). Wang’s study demonstrated that a high dose of *P. sibiricum* polysaccharide (PSP) significantly reduced body weight, FBG, fasting insulin (FINS), homeostasis model assessment-insulin resistance (HOMA-IR), and other parameters in STZ-induced diabetic rats (P < 0.05). In contrast, a low dose of PSP significantly reduced body weight and other parameters (P < 0.05) but did not have a significant effect on blood glucose levels (34).

Based on the beneficial effects of PRP reported in the literature, we can hypothesize a potential mechanism of action on glycemic control. The hypothesis of action of PRP is that it causes insulin-like effects by regulating the insulin signaling pathway (62). It is thus enticing to suggest that PRP confers beneficial effects on glucose homeostasis through the following pathways:

- It can promote glucose uptake by up-regulating the expression of glucose transporter 4 (GLUT-4) (62). Conversely, insulin resistance (IR) impedes glucose uptake by suppressing GLUT-4 expression (63). In addition, HFD-induced IR inhibits glucose uptake and adipocyte proliferation by inducing adipocyte inflammation. Therefore, inflammation inhibits cell survival and induces insulin resistance, and insulin resistance inhibits glucose uptake by reducing GLUT-4 expression (20).
- By regulating intestinal flora and short-chain fatty acids (SCFAs) it reduces low-grade inflammation, so as improves T2DM (64, 65). Early studies have indicated an association between impaired glucose metabolism and a modified ratio between the two primary phyla of human gut species, Firmicutes and Bacteroidetes (66, 67). In comparison with healthy individuals, diabetic patients exhibit a notable increase in the abundance of Firmicutes within the gut microbiota, alongside a significant decrease in the presence of Bacteroidetes (26, 68).
- It can mitigate islet cell damage, inhibit apoptosis of islet β cells, ameliorate insulin resistance, and enhance insulin expression and secretion (33) (46). Caspase-3, a vital member of the Caspase family, is activated by upstream initiator Caspases and subsequently targets specific substrates, inducing cellular and morphological alterations that culminate in apoptosis (69, 70). PSP exerts protective effects on islet cells by suppressing the expression of Caspase-3 protein, consequently decreasing β cell apoptosis and facilitating the hypoglycemic effect (71).
- It can mitigate inflammatory responses and oxidative stress damage (22) (45). The onset of T2DM is closely associated with elevated levels of pro-inflammatory cytokines (72). PRP can activate the Nrf2/HO-1 pathway and upregulate Nrf2 expression, thereby decreasing the levels of inflammatory factors interleukin-1β (IL-1β), interleukin-6 (IL-6), tumor necrosis factor-α (TNF-α), and C-reactive protein in mouse embryonic fibroblasts (3T3-L1) induced by high glucose and high insulin (20, 50). Advanced glycation end products (AGEs) augment the generation of reactive oxygen species and impair the antioxidant system, contributing to chronic stress in diabetes patients. PSP significantly curtails the production of AGEs, diminishes plasma malondialdehyde (MDA) levels, and suppresses superoxide dismutase activity, thereby thwarting oxidative stress (25, 49, 73).

**Figure 2:**
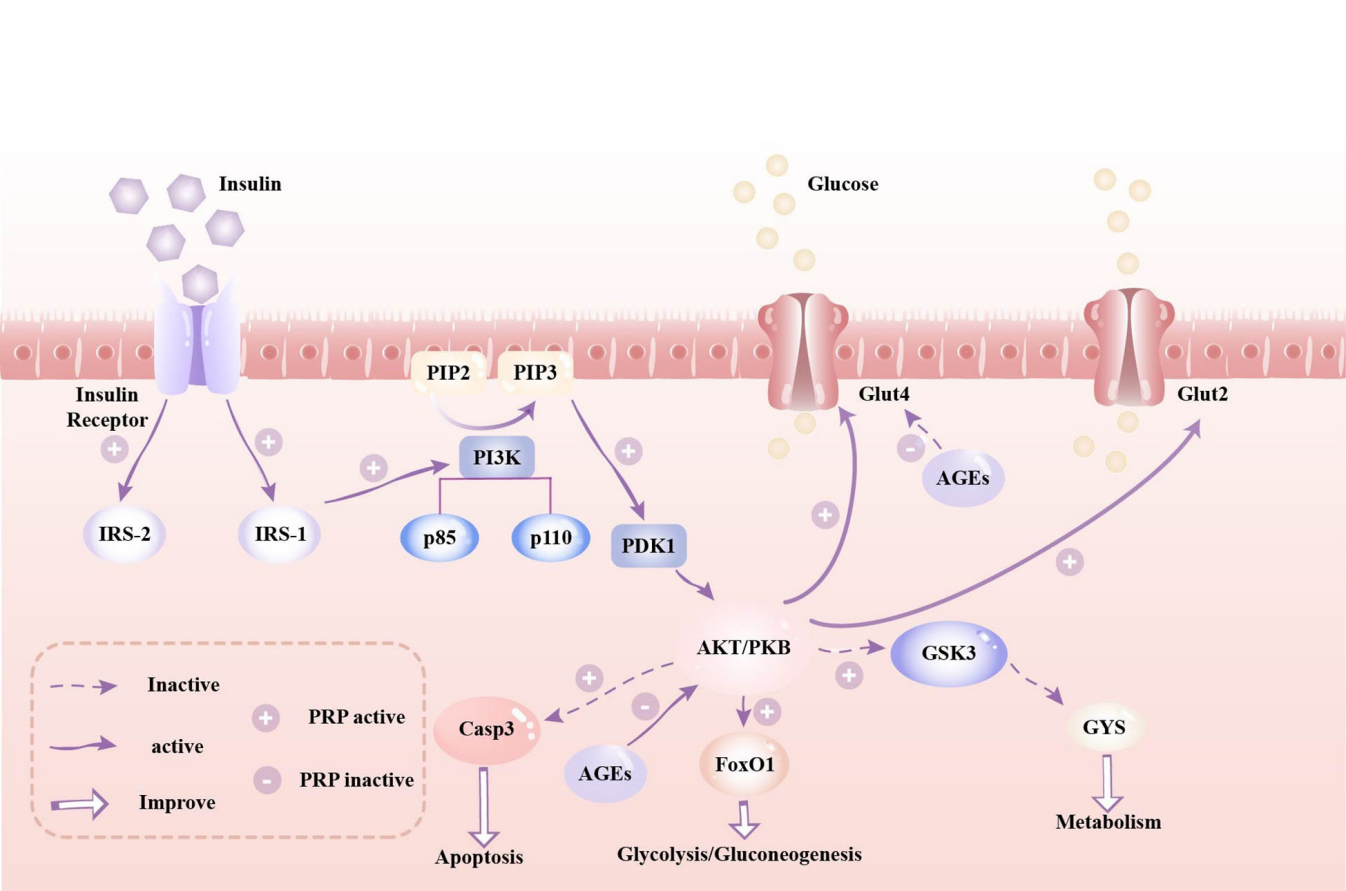
A schematic model depicting the mechanism(s) of action of *Polygonati Rhizoma* Polysaccharides in glycemic control. Legend: IRS, insulin receptor substrate; PIP2, phosphatidylinositol-4,5-bisphosphate; PIP3, phosphatidylinositol 3,4,5-trisphosphate; PI3K, Phosphatidylinosittol 3-kinases; PDK1, 3-phosphatidylinositide-dependent protein kinase 1; AKT/PKB, protein kinase B; Glut2, glucose transporter 2; Glut4, glucose transporter 4; Casp9, Caspase 9; Casp3, Caspase 3; FoxO1, Forkhead box O1; GSK3, Glycogen synthase kinase 3; GYS, Glycogen synthase.

Insulin plays a pivotal role in preserving blood glucose equilibrium, with the PI3K/AKT signaling pathway recognized as its primary target for blood glucose regulation (74). PRP modulates the insulin signaling pathway by initiating a cascade of events within the cell. This mechanism entails the activation and coordination of multiple biochemical steps to precisely regulate insulin signaling. PRP has demonstrated the ability to up-regulate the mRNA expression of PI3K, AKT, IRS1, IRS2, PDK1, GLUT2, and GYS (31, 33, 60). By activating the IRS1-PI3K-PDK1-AKT pathway, PRP enhances insulin secretion and facilitates the binding of insulin to insulin receptors, thereby enhancing the body’s glucose utilization capacity (30, 31, 75). Consequently, it can be inferred that PRP potentially induces conformational changes in the insulin receptor, leading to its phosphorylation by tyrosine kinase (PTK), consequently activating the insulin receptor substrate IRS. This activation initiates a cascade of downstream signaling molecules, including phosphatidylinositol kinase (PI3K)/protein kinase B (AKT/PKB), which suppresses downstream glycogen synthase activity, promotes glycogen synthesis, and enhances glucose transport (31, 76, 77). FoxO1 serves as an AKT-mediated substrate in the PI3K/AKT signaling pathway, and its activation correlates with insulin resistance and hyperglycemia (78). It has been proposed that AKT activation hinders the phosphorylation of FoxO1 and GSK3β, thereby suppressing gluconeogenesis and fostering glycogen synthesis (79, 80). Xie’s study (32) demonstrated that oral administration of PSPW (a homogeneous polysaccharide) significantly elevated the phosphorylation levels of PI3K and AKT, effectively attenuating the increase in phosphorylation of FoxO1 and GSK3β in T2DM model rats. Consequently, it can also be inferred that PSPW may curb gluconeogenesis and enhance glycogen synthesis by activating the insulin-mediated PI3K-AKT-FoxO1/GSK3 signaling pathway, thereby ameliorating insulin resistance and hyperglycemia. Caspase-3 protein serves as an AKT-mediated substrate within the PI3K/AKT pathway, and its activation can induce apoptosis (81). PRP has been shown to decrease blood glucose levels and enhance insulin expression in T2DM rats. This effect may be attributed to the ability of high-dose PRP to enhance AKT phosphorylation, down-regulate Caspase-3 expression, and inhibit apoptosis of islet cells (71, 82). Advanced glycation end products (AGEs) reduce insulin synthesis in islet beta cells by modulating FOXO activity (49), and they decrease muscle glucose uptake by inhibiting GLUT4 (83). Zhao’s study indicates that various PRPs can significantly inhibit AGE activity in a dose-dependent manner. The strongest inhibitory activity was observed at a concentration of 3 mg/mL, with an AGE inhibition rate of 30.2% (73). Similarly, Dong demonstrated that PRP enhances glucose uptake in skeletal muscle cells by upregulating GLUT-4 mRNA expression, thereby reducing blood glucose levels in STZ/HFD-induced SD rats (62). **Figure 2** depicts a schematic model elucidating the mechanism of action of PRP in glycemic control.

### 4.2 Molecular mechanisms of lipid regulation induced by *Polygonati rhizoma* polysaccharide

Different animal experiments have demonstrated that PRP can have beneficial effects on blood lipid profiles. PSP has been shown to significantly improve TC and TG levels, as well as reduce body weight in T2DM rats. In particular, a high dose of 800 mg/kg significantly decreased LDL-C levels, while various doses were found to significantly increase HDL-C levels in T2DM rats (37). Furthermore, a dose of PCP up to 260 mg/(kg· day) can significantly reduce serum TC, TG, and LDL-C levels, while increasing HDL-C levels in HFD-induced hyperlipidemic mice (35). Yue’s study demonstrated that PKP can improve lipid metabolism disorders by activating peroxisome proliferator-activated receptor gamma (PPARγ) in adipocytes and inhibiting the Toll-like receptor 4/nuclear factor kappa B (TLR4/NFκB) signaling pathway. Additionally, the water extract of PK showed a significant regulatory effect on endogenous metabolites in rats with lipid metabolism disorders (29).

**Figure 3:**
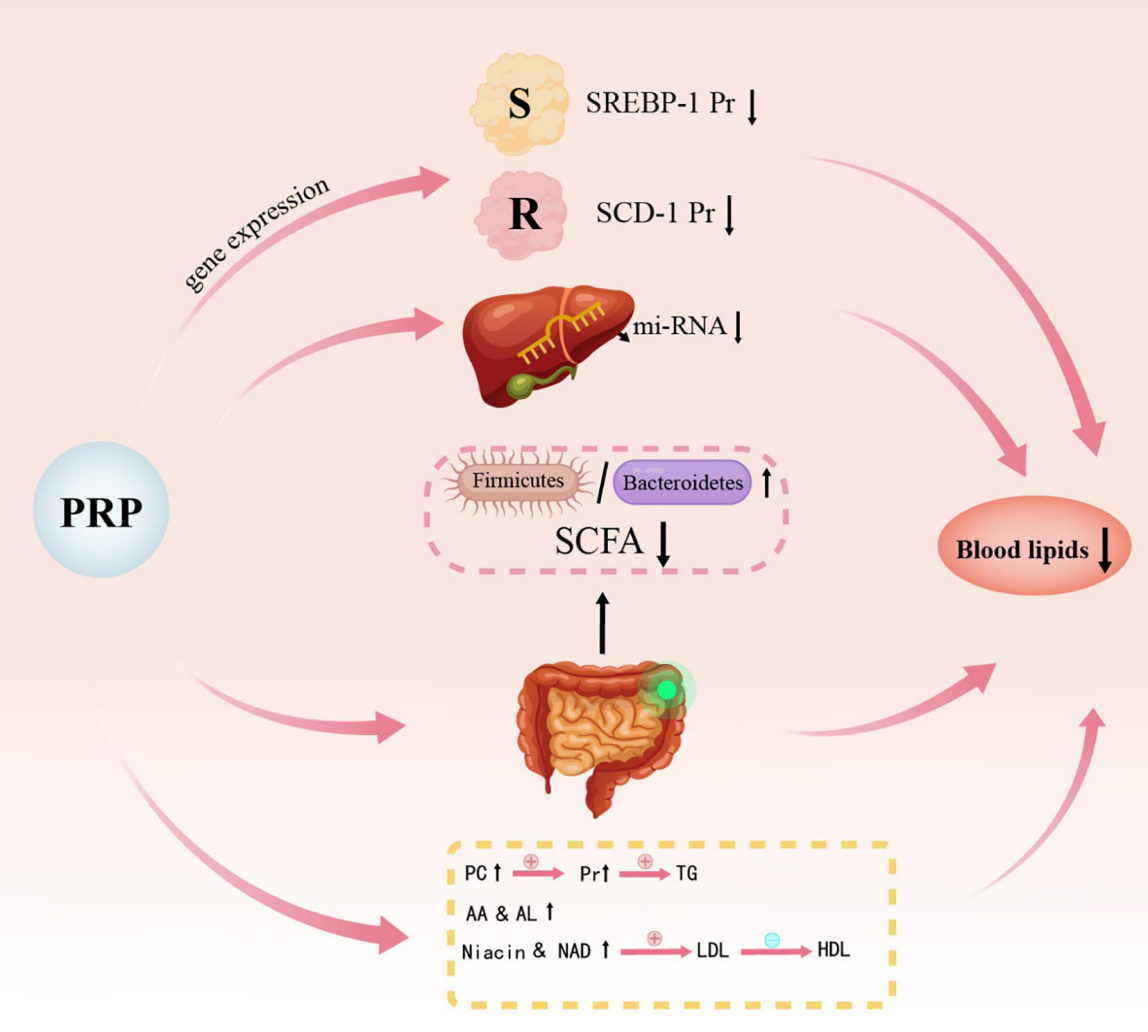
Schematic diagram illustrating the mechanism of action of *Polygonati Rhizoma* Polysaccharide on lipid metabolism. SCD-1, Stearoyl-CoA desaturase 1; PC, Phosphatidylcholine; AA, Arachidonic acid; LA, Linoleic acid; NA, Nicotinic acid; Nam, Niacinamide.

**Figure 4:**
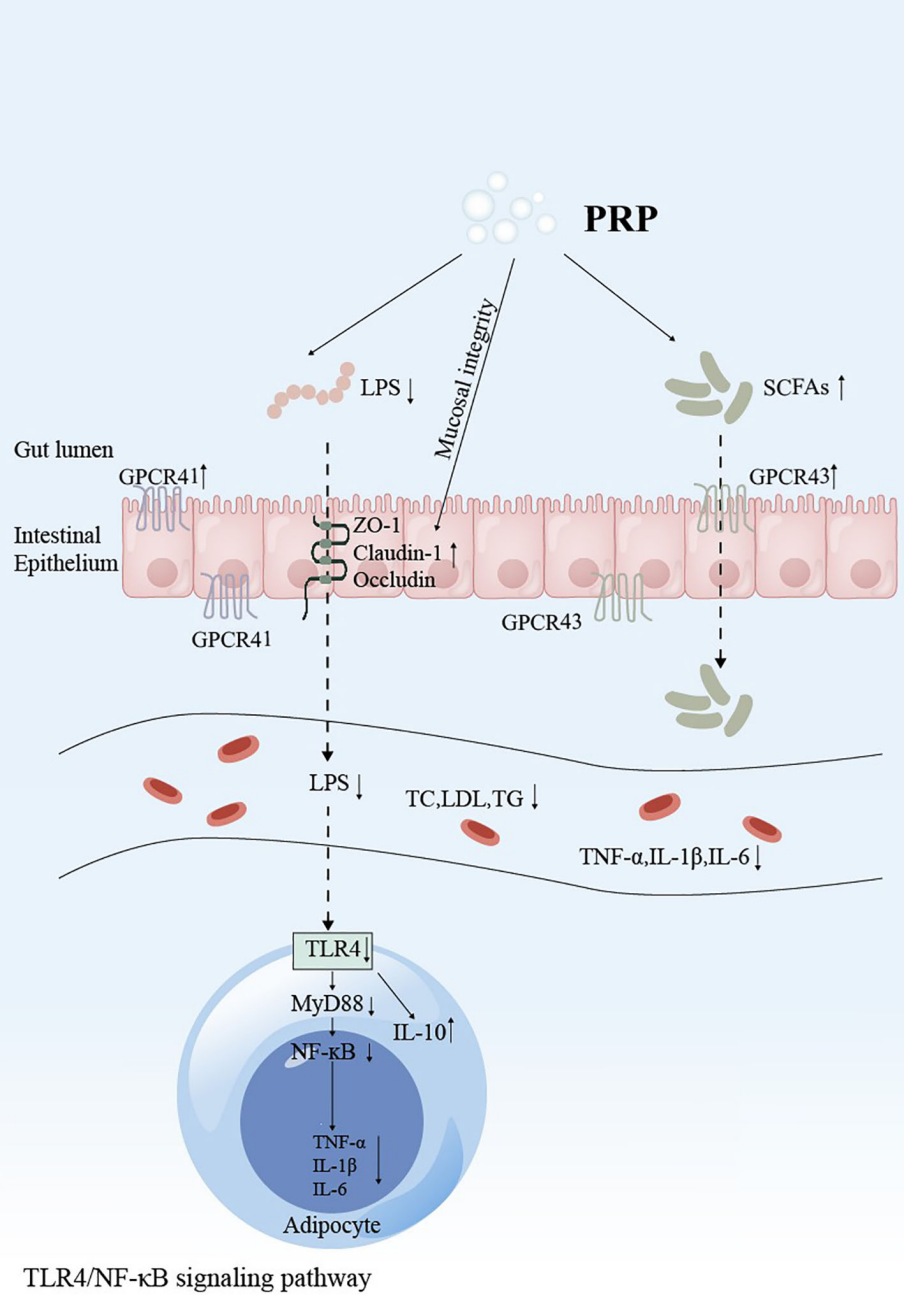
Schematic representation of *Polygonati Rhizoma* Polysaccharide intervention in the TLR4/MyD88/NF-κB signaling pathway. GPCRs, G protein-coupled receptors; ZO-1, Zona Occludens 1; LPS, Lipopolysaccharide; TNF-α, Tumor necrosis factor-α; IL-1β, Interleukin-1β; IL-6, Interleukin-6; IL-10, Interleukin-10; TLR4, Toll-like receptor 4; MyD88, Myeloid differentiation primary response 88; NF-κB, Nuclear factor kappa-light-chain-enhancer of activated B cells.

The mechanism by which PRP regulates lipid metabolism has been rarely studied in the literature. However, the mechanism of PRP’s effects on T2DM rats can be hypothesized based on existing studies. **Figures 3** and **4** illustrate the potential pathways and mechanisms by which PRP may lower blood lipid levels in T2DM rats.

PRP exerts lipid-lowering effects through several key mechanisms. Peroxisome proliferator-activated receptors (PPARs) have diverse biological functions, primarily in regulating adipocyte gene expression, promoting adipocyte differentiation, and improving insulin resistance (84). Sterol regulatory element-binding proteins (SREBPs) are nuclear transcription factors that play a key role in the negative feedback regulation of cholesterol metabolism (85). One subtype, SREBP-1c, is involved in both fatty acid and glucose metabolism and is a major transcriptional regulator of lipogenic genes, primarily influencing fatty acid synthesis and triacylglycerol accumulation (86). Studies have demonstrated that the expression levels of PPARα, PPARβ/δ, and PPARγ are upregulated in HFD rats fed with PRP (26, 29, 45, 87), while the expression levels of SREBP-1c and stearoyl-CoA desaturase 1 (SCD-1) are downregulated (37, 59). These findings suggest that PRP may reduce lipid levels by modulating the PPARs/SREBP-1c signaling pathway.

Yang’s study on the non-targeted metabolomics of *P. kingianum* water extract, which primarily contains polysaccharides and saponins, demonstrated that *P. kingianum* alleviates HFD-induced dyslipidemia by regulating endogenous metabolites in serum, urine, and liver samples. The identified biomarkers include those involved in the biosynthesis of phenylalanine, tyrosine, tryptophan, valine, leucine, and isoleucine, as well as the metabolism of tryptophan, tyrosine, phenylalanine, starch, sucrose, glycerolipids, arachidonic acid, linoleic acid, niacin, niacinamide, and sphingolipids (88).

Current studies on PRP in reducing blood lipids primarily highlight its ability to mitigate lipid metabolism disorders by inhibiting the TLR4/NF-κB signaling pathway. The tight junctions of the small intestinal mucosal epithelia in HFD-fed rats were significantly damaged and necrotic. The expression of the tight junction proteins (TJPs) ZO-1, Claudin-1, and occludin was markedly lower than in normal rats, leading to the entry of lipopolysaccharides (LPS) into the bloodstream. LPS is recognized by lipopolysaccharide-binding protein (LBP), forming a complex that transfers to CD14, activates the transmembrane protein TLR4, and subsequently promotes the activation of NF-κB, which regulates the expression of inflammatory factors (89). PRP can reverse intestinal barrier damage induced by HFD. It increases the expression of the TJPs ZO-1, Claudin-1, and Occludin in HFD-fed rats, enhances the expression of SCFA receptors GPCR41 and GPCR43, and down-regulates the expression of TLR4 and MyD88 (29, 35, 90, 91). By strengthening the intestinal barrier integrity, PRP reduces serum LPS levels, inhibits the release of pro-inflammatory factors IL-6, IL-1β, and TNF-α mediated by the TLR4/NF-κB signaling pathway, and promotes the production of the anti-inflammatory factor IL-10 (26, 45, 87). This relieves chronic low-grade inflammation and improves lipid metabolism disorders.

### 4.3 *Polygonati rhizoma* polysaccharide modulates gut microbiota to improve glucose and lipid metabolism

Research by Qin identified moderate gut microbial dysbiosis as a hallmark of T2DM, marked by decreased levels of prevalent butyrate-producing bacteria, expansion of opportunistic pathogens, and enrichment of microbial functions associated with sulfate reduction and oxidative stress resistance. Mounting evidence supports a strong link between the gut microbiota and diabetes pathogenesis (92–94). Consequently, some researchers have shifted their focus to the potential of active components from traditional Chinese herbs to exert their effects via microbiota-mediated pathways.

MicroRNAs (miRNAs) are small non-coding RNAs that regulate gene expression by silencing target mRNAs (95). Increasing evidence suggests that miRNAs play a significant role in lipid metabolism and related diseases (96, 97). MiR-122, the first miRNA identified to be associated with lipid metabolism, is specifically expressed in the liver and accounts for approximately 70% of all liver mRNAs (98). Research by Dong found that the total polysaccharide (PS) and high molecular weight polysaccharide fraction (PSF) from *P.kingianum* can regulate the composition, abundance, and diversity of intestinal microbiota in HFD rats. These treatments increase the relative abundance of SCFA-producing bacteria, enhance SCFA production, reduce intestinal permeability, alleviate gastrointestinal inflammation, and improve lipid metabolism (26). PSF was found to significantly downregulate miR-122 in the liver, leading to reduced expression of downstream genes involved in lipid synthesis. In HFD-induced obese mice, miR-484 levels were significantly reduced but were restored following treatment with PS, PSF, and simvastatin. Additionally, the abundance of *Roseburia* in HFD-induced obese mice was also restored after these treatments. Consequently, Dong proposed that the miR-484-*Bacteroides*/*Roseburia* axis is a critical factor in the therapeutic effects of *P. kingianum* (99).

The intestinal structure plays a crucial role in maintaining the function of the intestinal barrier, and intestinal microbes are key factors influencing metabolic diseases. Alterations in the community structure of these microbes can result in metabolic dysfunction. The composition of the intestinal flora is closely associated with diabetes, and its regulation can help alleviate symptoms and reduce mucosal inflammation to a certain extent (100). As essential signaling molecules, SCFAs provide energy for intestinal cells, regulate their growth and differentiation, and help maintain the integrity of the epithelial barrier (101). Studies have shown that PRP can increase the relative abundance of SCFA-producing bacteria and promote SCFA production (26, 64, 87, 90). SCFAs can inhibit the NF-κB signaling pathway, thereby reducing intestinal inflammatory responses and improving lipid metabolism disorders (102). In comparison to normal rats, HFD-induced rats exhibit a lower relative abundance of *Bacteroidetes* and a higher relative abundance of *Firmicutes*. Notably, the ratio of *Firmicutes* to *Bacteroides* (F/B ratio) plays a crucial role in metabolic processes and is positively correlated with body mass index (BMI) (103). HFD-fed rats display reduced gut microbiota diversity and a significant increase in the F/B ratio. However, treatment with PRP led to an increase in both the abundance and diversity of intestinal flora in the model rats, along with a reduction in the F/B ratio (26, 68, 87, 90).

Furthermore, studies demonstrate that *Akkermansia muciniphila* can counteract metabolic disorders induced by a high-fat diet, including increased adiposity, metabolic endotoxemia, adipose tissue inflammation, and insulin resistance, while also enhancing gut microbiota diversity (104, 105). Research by Luo showed that PSP-1, a monomeric polysaccharide isolated and purified from *Polygonatum sibiricum*, restores gut microbiota composition. Furthermore, it decreases the relative abundance of *Helicobacter* spp. while enhancing *Akkermansia muciniphila* abundance, leading to the indirect attenuation of hyperglycemia and hyperlipidemia associated with diabetes (106). Zhang demonstrated that *Polygonatum sibiricum* polysaccharide (PSP) exerts anti-hyperglycemic, anti-inflammatory, and antioxidant effects in T2DM via: (1) Gut microbiota restoration; (2) Modulation of serum metabolic pathways (arginine-proline, tryptophan, and glutathione metabolism) (28).

Beyond PRPs, numerous studies have demonstrated the ability of herbal polysaccharides to ameliorate diabetes via modulation of the gut microbiota. Song revealed that *Astragalus membranaceus* polysaccharide (AMP) treatment increases SCFA production. This elevation stimulates glucagon-like peptide-1 (GLP-1) secretion and enhances intestinal barrier integrity by upregulating GPCR41/43 and TJPs (Occludin and Zonula Occludens-1 (ZO-1)). These collective effects contribute to the alleviation of diabetic symptoms in db/db mice (107). Chen investigated the effects of *Ganoderma lucidum* polysaccharide (GLP) on the gut microbiota and fecal metabolites in T2DM rats. Their study found that GLP effectively restored dysbiotic gut microbiota to a healthier state. This restoration was characterized by a significant reduction in the abundance of potentially harmful bacteria, including *Aerococcus*, *Ruminococcus*, *Corynebacterium*, and *Proteus*, alongside an increase in beneficial genera such as *Blautia*, *Dehalobacterium*, *Parabacteroides*, and *Bacteroides*. Concomitant modulation of host metabolites underlies GLP’s anti-diabetic efficacy (108).

## 5 Discussion

Within the theoretical framework of Traditional Chinese Medicine (TCM), “Xiaoke” mirrors modern diabetes mellitus, characterized by polydipsia, polyuria, polyphagia, and weight loss (109). *Huang Jing* (Polygonati Rhizoma, PR), a cornerstone TCM herb for Xiaoke, has garnered significant scientific interest for its polysaccharides in modulating glucose and lipid metabolism in T2DM. This review synthesizes research from the past 15 years, highlighting PRP’s substantial potential in ameliorating hyperglycemia and dyslipidemia in T2DM models. The non-toxic nature and abundance of these polysaccharides have positioned them as promising candidates for further exploration. The accumulated evidence indicates that PRP supplementation effectively reduces blood glucose, improves lipid profiles, mitigates inflammation, and alleviates associated complications in diabetic rodent models.

The hypoglycemic action primarily involves direct modulation of insulin signaling pathways. PRP activates PI3K/AKT in hepatic and skeletal muscle tissues, enhancing glucose uptake and glycogen synthesis (30–34, 75). This is evidenced by reduced FBG, HbA1c, and HOMA-IR in diabetic models, alongside upregulated GLUT4 expression and IRS/PI3K/AKT phosphorylation (62, 75). Concurrently, PRP suppresses gluconeogenesis via PEPCK1 downregulation (110) and mitigates insulin resistance through Nrf2/HO-1-mediated antioxidant pathways (20, 50, 52).

For lipid regulation, PRP operates largely via gut microbiota remodeling. It enriches beneficial genera (e.g., *Bifidobacterium*, *Lactobacillus*) while reducing pathogenic bacteria (e.g., *Prevotella*), thereby elevating SCFA production (especially butyrate) and strengthening intestinal barrier integrity (ZO-1/occluding ↑) (26, 28, 64, 68, 87, 111). This suppresses LPS-induced TLR4/NF-κB inflammation and activates GPR41/43 signaling, collectively improving lipid profiles (↓TG, TC, LDL-C; ↑HDL-C) (87, 90). Hepatic lipid metabolism is further normalized via AMPK/SREBP-1c inhibition and PPARα activation (37, 45, 59, 112).

Beyond metabolic endpoints, PRP demonstrates multi-organ protective effects. It alleviates diabetic nephropathy by inhibiting TGF-β1/Smad2/3 fibrotic pathways and ferroptosis (39–41, 113, 114), shields retinal cells via anti-apoptotic mechanisms (Bcl-2↑/Bax↓) (25, 61), and preserves testicular function through autophagy activation (115).

Different species of *Polygonatum* polysaccharides exert hypoglycemic and hypolipidemic effects through distinct mechanisms. *P. sibiricum* polysaccharides primarily activates intracellular signaling pathways, such as PI3K/AKT, to promote glucose uptake, inhibit gluconeogenesis, and regulate lipid metabolism (30, 32, 34, 75). It modulates the gut microbiota structure and metabolic pathways, altering microbial composition, regulating serum metabolites, and influencing the Microbiota-SCFA axis to improve metabolic health (28, 68, 111). Lipid synthesis is suppressed via the AMPK/SREBP-1c pathway, downregulating key lipogenic genes (*SREBP-1c*, *SCD-1*, *FAS*) (37, 45, 59, 112). Additional protective mechanisms include antioxidant and anti-inflammatory activities, notably through activation of the Nrf2/HO-1 axis (20). *P. cyrtonema* polysaccharides activates the T1R2/T1R3-Gαs-PKA-PC3 axis to stimulate GLP-1 secretion and improve glucose tolerance, thereby lowering blood glucose (116). It also alleviates oxidative stress and inflammation by activating the Nrf2/HO-1 axis and suppressing pro-inflammatory signaling pathways (35, 50). Modulation of intestinal signaling pathways, the gut barrier, and microbiota composition contributes to its metabolic effects (117). Furthermore, it exerts comprehensive regulation of glucose and lipid metabolism through mechanisms including upregulation of osteogenic gene expression and hepatoprotection (118). *P.kingianum* polysaccharides directly enhances glucose utilization and modulates lipid profiles by activating the PI3K/AKT pathway and promoting β-cell repair and regeneration (33, 119, 120). Indirectly, it influences metabolism by regulating gut microbiota-related axes, such as the Gut microbiota-SCFA axis and miR-484 signaling (26, 64, 90, 99). Additionally, it achieves hypoglycemic and hypolipidemic effects by modulating multiple metabolic pathways, mitigating oxidative stress, and reducing levels of inflammatory and fibrotic factors, thereby maintaining metabolic homeostasis (39, 52, 88, 121).

Despite robust preclinical evidence, critical gaps persist. Despite substantial preclinical evidence supporting PRP’s efficacy, critical knowledge gaps remain. First, there is a notable paucity of clinical validation for PRP’s therapeutic benefits in T2DM. Most existing studies rely on rodent models (e.g., HFD/STZ-induced rats), and while PR has been investigated as a component of Chinese herbal formulae, dedicated clinical trials are urgently needed to confirm its efficacy and safety profile. Second, Once the topic of polysaccharides is discussed, the study of structure-activity relationships cannot be avoided. Structural heterogeneity—molecular weight, monosaccharide composition, and glycosidic linkages—across *P. kingianum*, *P. sibiricum*, and *P. cyrtonema* sources complicates activity comparisons (117, 122). For instance, the polysaccharide (PSP) isolated from *P. sibiricum* exhibits enhanced phagocytic activity and stronger DPPH free radical scavenging capacity when it has a higher molecular weight. In contrast, low-molecular-weight PSP demonstrates greater efficiency in eliminating ABTS^+^ and hydroxyl free radicals (123, 124). From the perspective of monosaccharide composition, PSP shows stronger antioxidant activity when it has a lower molar ratio of fructose to glucose but a higher molar content of arabinose (53). For *P. cyrtonema* polysaccharide (PCP) with specific functional group structures, its immunomodulatory activity is positively correlated with the content of O-acetylated fructan—the higher the content, the stronger the activity (125). The biological activity of polysaccharides is closely related to their structure. These features include molecular weight, monosaccharide composition, and glycosidic bonds. Studying the structure-activity relationship of PRP is crucial for elucidating its mechanism of action and improving clinical efficacy. Third, optimal dosing remains undefined; effective doses range widely (e.g., 5–10 g/kg in rats) (62, 126), hindering clinical translation. Future directions: standardized PRP isolates, mechanistic dose-response studies, and randomized controlled trials (RCTs) are needed to integrate TCM theory with evidence-based medicine.

## 6 Conclusion

PRP ameliorates T2DM by targeting hyperglycemia and dyslipidemia. Its hypoglycemic effect relies on activating the PI3K/AKT pathway to enhance insulin sensitivity, promote glucose transport, and reduce blood glucose. For lipid regulation, PRP modulates gut microbiota (increasing SCFA-producing bacteria), strengthens intestinal barrier function, suppresses the TLR4/MyD88/NF-κ B pathway, and regulates hepatic lipid factors (PPARs, SREBP-1c). It also mitigates inflammation/oxidative damage via Nrf2/HO-1, protecting kidneys and retina.

Clinical use is limited by lacking human trials, structural heterogeneity across *Polygonatum* species, and undefined optimal dosages. In summary, PRP is a promising multi-target candidate for T2DM therapy, integrating glucose-lowering, lipid-regulating, and anti-inflammatory actions. Future research needs standardized, randomized clinical trials, determination of optimal dosages, scalable extraction protocols, and structural-activity studies to bridge traditional use with modern pharmacology and realize its full therapeutic potential.
