# Supplementary material for: Complementary efficacy and molecular insights of Polygonati rhizoma polysaccharide for glycemic and lipid abnormalities in type 2 diabetes mellitus: a comprehensive review
Source: Front Endocrinol (Lausanne). 2025 Sep 10;16:1606816. doi: 10.3389/fendo.2025.1606816 (PMC12457904; doi:10.3389/fendo.2025.1606816)
Supplement: Supplementary file 1 [file Table1.doc]

Complementary Efficacy and Molecular Insights of *Polygonati Rhizoma* Polysaccharide for Glycemic and Lipid Abnormalities in Type 2 Diabetes Mellitus: A Comprehensive Review

Shichao Ma^1^, Lu Xu^1^, Yadi Hou^1^, Wenjing Zhang^1^, Yongxia Cui^1, *^, Suiqing Chen^1,2,3, **^

^1^ College of Pharmacy, Henan University of Chinese Medicine, Zhengzhou, 450046, China

^2^ Henan Provincial Key Laboratory of Chinese Medicine Resources and Chinese Medicine Chemistry, Henan University of Chinese Medicine, Zhengzhou, 450046, China

^3^ Henan University of Chinese Medicine, Collaborative Innovation Center of Research and Development on the Whole Industry Chain of Yu-Yao, Henan Province 450046, China

**Table S1. Complete Literature Screening List.**

| **NO.** | **References** | **Key Effect** | **Species** | **Mechanism** | **Model System** | **Key Pathway/Targets** | **Outcome** | **Title** |
| --- | --- | --- | --- | --- | --- | --- | --- | --- |
| 1 | Li et al.(1) | Glucose Reduction | *P.kingianum* | Direct | **In vivo:** STZ-induced diabetic mice **In vitro：**HepG2 hepatocytes | PI3K/AKT activation | ↓ FBG, ↑ Insulin sensitivity | Structural characterization, hypoglycemic effects and antidiabetic mechanism of a novel polysaccharides from *Polygonatum kingianum* Coll. et Hemsl |
| 2 | Gu et al.(2) | Glucose & Lipids Reduction | *P.kingianum* | Indirect | HFD-induced dyslipidemic rats | Gut microbiota-SCFA axis | ↓ HbA1c, ↑ HDL-C/LDL-C ratio | Polysaccharides from *Polygonatum kingianum* improve glucose and lipid metabolism in rats fed a high fat diet |
| 3 | Cai et al.(3) | Glucose Reduction | *P. sibiricum* | Direct & Indirect | IR-3T3-L1 adipocytes | Nrf2/HO-1 axis activation | ↓ TNF-α/IL-6, ↑ GLUT4 | *Polygonatum sibiricum* polysaccharide alleviates inflammatory cytokines and promotes glucose uptake  in high‑glucose‑ and high‑insulin‑induced 3T3‑L1 adipocytes by promoting Nrf2 expression |
| 4 | Cai et al.(4) | Glucose & Lipids Reduction | *P. sibiricum* | Direct | PA-induced IR L6 myotubes | miR-340-3p/IRAK3 axis | ↑ Glucose uptake, ↓ TG, ↓ miR-340-3p | *Polygonatum sibiricum* polysaccharides (PSP) improve the palmitic acid (PA)-induced inhibition of survival, inflammation, and glucose uptake in skeletal muscle cells |
| 5 | Wang et al.(5) | Glucose & Lipids Reduction | *P. sibiricum* | Direct | HFD/STZ-induced T2DM mice | PI3K/AKT activation | ↓ FBG/HbA1c; ↓ TC/TG/LDL-C; ↓ Hepatic TG; ↑ Insulin sensitivity | Aqueous extract of *Polygonatum sibiricum* ameliorates glucose and lipid metabolism via PI3K/AKT signaling pathway in high‐fat diet and streptozotocin‐induced diabetic mice |
| 6 | Yan et al.(6) | Glucose Regulation | *P.kingianum* | Indirect | HFD/STZ-induced T2DM rats | Gut microbiota remodeling (↑ *Firmicutes***, ↓** *Bacteroidetes*) | ↓ FBG; ↑ Fecal SCFAs (Butyrate↑); ↑ *Ruminococcaceae*; ↓ LPS (Endotoxemia) | Intake of total saponins and polysaccharides from *Polygonatum kingianum* affects the gut microbiota in diabetic rats |
| 7 | Zhang (7) | Glucose Regulation & Renal Protective | *P. sibiricum* | Direct & Indirect | STZ-induced diabetic SD rats | ↓ Blood glucose ↓ Renal AgII (RAS suppression) ↓ MDA (antioxidant) ↓ AGEs-mediated injury | ↓ Fasting glucose (P<0.01 vs. model); ↓ UMA/SCR/BUN (P<0.05); ↓ Renal AgII/MDA (P<0.05); ↑ Body weight (P<0.01); ↓ Kidney/body weight ratio (P<0.05); Key limitation: Weaker AgII/SCR/BUN reduction than captopril P<0.05) | The therapeutic effect of *polygonatum sibiricum* polysaccharid on DN rats and the possible mechanism |
| 8 | Jia et al.(8) | Glucose Reduction | *P. sibiricum* | Direct | Long-term HFD-induced T2DM mice (50-week feeding) | IRS-2 upregulation ↓ Hepatic NO | ↓ FBG & Fasting Insulin; ↑ IRS-2 expression; ↑ Insulin Sensitivity;  Improved oral glucose tolerance | Effects of *Polygonati Rhizoma* Polysaccharide on glucose metabolism in diabetic mice induced by high fat diet |
| 9 | Wang et al.(9) | Glucose & Lipids Reduction | *P. sibiricum* | Direct | HFD/STZ-T2DM mice | Hepatic PI3K/AKT signaling ↑ | ↓ FBG/FINS/HOMA-IR; ↓ TC/TG/LDL-C/hepatic TG; Improved hepatic steatosis (dose-dependent); ↑ PI3K/AKT mRNA | Study on the prevention of glyeolipid metabolism disorder in mice by *Polygonatum sibiricum* polysaccharides |

**Table S1.** Cont

| **NO.** | **References** | **Key Effect** | **Species** | **Mechanism** | **Model System** | **Key Pathway/Targets** | **Outcome** | **Title** |
| --- | --- | --- | --- | --- | --- | --- | --- | --- |
| 10 | Xie et al.(10) | Glucose Reduction | *P. sibiricum* | Direct | HFD/STZ-induced T2DM mice | PI3K/AKT phosphorylation + GLP-1 secretion | ↓FBG/GSP;  ↑Insulin sensitivity; ↑GLP-1 | Physicochemical characterization and hypoglycemic potential of a novel polysaccharide from *Polygonatum sibiricum* Red through PI3K/Akt mediated signaling pathway |
| 11 | Zhang et al.(11) | Glucose Reduction & Anti-inflammatory/Antioxidant | *P. sibiricum* | Indirect | HFD/STZ-induced T2DM mice | ↓ *Firmicutes/Bacteroidetes* ratio; ↑*Blautia/Adlercreutzia/Akkermansia;* ↓*Prevotella/Megamonas/Escherichia;* Regulates serum metabolites in:  Arginine/proline metabolism;  Tryptophan metabolism;  Glutathione metabolism; | ↓ Fasting glucose (P<0.05) ↓ Inflammation/oxidative stress; Microbiome:  F/B ratio ↓, beneficial genera ↑; Metabolomics: 20 altered metabolites (e.g., ↑ glutathione, ↓ kynurenine) | Integrated 16S rRNA Sequencing and Untargeted Metabolomics Analysis to Reveal the Protective Mechanisms of *Polygonatum sibiricum* Polysaccharide on Type 2 Diabetes Mellitus Model Rats |
| 12 | Teng et al.(12) | Glucose Reduction & Antioxidant | *P. cyrtonema* | Indirect | *In vivo*:Oxidative damage mice In vitro:Free radical assays | Nrf2/HO-1 activation | ROS↓, Enzymes↑→↑ Insulin sensitivity | Polysaccharides from steam‐processed *Polygonatum cyrtonema* Hua protect against d‐galactose‐induced oxidative damage in mice by activation of Nrf2/HO‐1 signaling |
| 13 | Yang(13) | Glucose Reduction | *P. cyrtonema* | Indirect | Rat intestine/NCI-H716 cells | T1R2/T1R3-Gαs-PKA-PC3 axis | ↑Portal GLP-1, ↑Glucose tolerance | The Effect of *Polygonatum Cyrtonema* Hua Polysaccharides（PCP） on the Expression and Secretion of GLP-1 and its Molecular Mechanism |
| 14 | Gao et al.(14) | Glucose Reduction | *P. sibiricum* | Indirect | α-Glucosidase assay | α-Glucosidase competitive inhibition | 68.1% enzyme inhibition (2-14 kDa glucan); Structure-activity relationship:  Glucose homopolymer > heteropolysaccharides | Extract of *Polygonatum sibiricum* and their inhibition of α-glucosidase |
| 15 | Wang et al.(15) | Glucose Reduction & Ocular Protection | *P. sibiricum* | Direct & Indirect | STZ-diabetic SD rats | ↓ Hyperglycemia (FBG/HbA1c↓) ↓ Oxidative stress (MDA↓; SOD↑) ↑ Insulin/C-peptide | ↓FBG/HbA1c; ↑Insulin/C-peptide (P<0.05 dose-dependent); ↓ Polydipsia/polyphagia/weight loss (P<0.05) | Original Research: Potential ocular protection and dynamic observation of *Polygonatum sibiricum* polysaccharide against streptozocin-induced diabetic rats' model |
| 16 | Zeng et al.(16) | Glucose & Lipids Reduction | *P. sibiricum* | Direct & Indirect | STZ-diabetic mice | IRS1-PI3K-PDK1-Akt axis ↑ PI3K-Akt-GSK3β-GYS pathway ↑ PI3K-Akt-PIP5K-GLUT2 axis ↑ Antioxidant defense ↑ (SOD/GSH-Px) | ↓ Blood glucose (day 7-28, p<0.01); ↓ TC/TG/MDA (p<0.01); ↑ Insulin/hepatic glycogen (p<0.01);  ↑ SOD/GSH-Px activities; GSY/GLUT2/PIP5K ↑ & GSK3β ↓ (mRNA & protein) | Hypoglycemic effect and mechanism of *Polygonatum sibiricum* polysaccharides on diabetic mice |
| 17 | Ren et al.(17) | Glucose Regulation | *P. sibiricum* | Indirect | HFD/STZ-T2D mice | Gut microbiota remodeling ↑ *Firmicutes****/****Bacteroidetes* ratio ↓ *Verrucomicrobia* Intestinal histopathology improvement | ↓ Blood glucose (26.6% HD);  ↑ Body weight (14.24% HD); Improved jejunal architecture;  Dose-dependent efficacy | Study on hypoglyeemie effeet and intestinal effeet of *Polygonatum sibiricum* polysaccharides in diabetic mice |

**Table S1.** Cont

| **NO.** | **References** | **Key Effect** | **Species** | **Mechanism** | **Model System** | **Key Pathway/Targets** | **Outcome** | **Title** |
| --- | --- | --- | --- | --- | --- | --- | --- | --- |
| 18 | Wang(19) | Glucose & Lipids Reduction | *P. sibiricum* | Direct | HFD/STZ-T2D mice | PI3K/AKT pathway activation ↑ IRS1/PI3K/AKT/GSK3β (mRNA & protein) ↑ p-IRS1/p-AKT/p-GSK3β (phosphorylation) | ↓ FBG; ↓ HbA1c; ↓ HOMA-IR; ↓ TG/LDL-C/TC; ↓ Hepatic steatosis;  ↑ Glycogen storage (PAS+); PSAE=PSP efficacy | The Protective Effects of *Polygonatum Sibricum* Aqueous Extract on Glycolipid metabolism in Mice Based on PI3K/AKT Pathway |
| 19 | Dong(20) | Glucose & Lipids Reduction | *P. sibiricum* | Direct | In vivo: HFD/STZ-T2DM rats In vitro: FFA-induced IR L02 hepatocytes | PINK1/Parkin mitophagy activation Mitochondrial quality control ROS-ATP homeostasis restoration | ↓ Hepatic steatosis (Oil Red O↓); ↑ ATP (2.1-fold) & ↓ ROS (62%); Mdivi-1 abolishes effects; p-PI3K/p-AKT ↑ | To explore the mechanism of Polygonum polygonum polysaccharide in alleviating T2DM based on mitophagy mediated by PINK1/Parkin |
| 20 | Liu et al.(21) | Glucose & Nephroprotective | *P.kingianum* | Indirect | STZ/HFD-induced diabetic nephropathy Kunming mice | ↓ Pro-inflammatory cytokines (TNF-α, IL-1β, IL-6) ↓ Fibrogenic factors (TGF-β1, Fibronectin) | ↓ FBG (P<0.05); ↓ 24h urine volume/protein (P<0.05); ↓ Serum creatinine (Scr)/BUN (P<0.05); ↓ Renal TNF-α/IL-1β/IL-6 (P<0.05); ↓ Renal TGF-β1/Fibronectin (P<0.05) | Effect of *Polygonatum Yunnanensis* Polysaccharide on Diabetic Nephropathy Mice Caused by Low Concentration of Streptozotocin |
| 21 | Sun(22) | Glucose Reduction & Testicular Protective | Unspecified species | Direct | STZ/HFD-induced T2DM SD rats | ↑ LKB1 expression ↓ p-mTOR/mTOR ratio ↑ p-ULK1/ULK1 ratio ↓ P62; ↑ ATG5 → Autophagy activation | ↓ FBG (P<0.05); ↑ Sperm concentration & motility; Improved testicular histology: Seminiferous tubule structure, spermatogenic cells ↓ P62; ↑ ATG5/LKB1 proteins (P<0.05) | Based on the theory of "Yin deficiency is often insufficient", a study on the improvement of reproductive damage in diabetic male rats by *Polygonati Rhizoma* through the AMPK-mTOR-ULK1 pathway was explored. |
| 22 | Dong et al.(23) | Glucose Reduction | Unspecified species | Direct | STZ/HFD-induced T2DM SD rats | ↑ GLUT-4 mRNA expression → Enhanced skeletal muscle glucose uptake | ↓ FBG (P<0.01); ↑ GLUT-4 mRNA in muscle tissue (P<0.01); Dose-dependency:  Optimal efficacy at 5.0-10.0 g/kg/d;  ↑Insulin sensitivity; | Effects of rhizoma polygonati on the expression of glucose transporter-4 gene in type 2 diabetes mellitus rats with insulin resistance |
| 23 | Chen et al.(24) | Glucose Reduction | *P.kingianum* | Direct | Adrenaline-induced stress hyperglycemia Alloxan-induced β-cell damage diabetes | Potential β-cell repair/regeneration Glucose utilization enhancement | Normoglycemia: No glucose reduction (P>0.05); Glucose challenge (10%): ↓ Glycemia (P<0.05); Adrenaline (240μg/kg): ↓ Glycemia (P<0.05); Alloxan (80mg/kg): ↓ Glycemia (P<0.01) | Experimental Investigation of *Polygonatum kingianum* on Blood Glucose in Induced Hyperglycemic Mice |
|  |  |  |  |  |  |  |  |  |

**Table S1.** Cont

| **NO.** | **References** | **Key Effect** | **Species** | **Mechanism** | **Model System** | **Key Pathway/Targets** | **Outcome** | **Title** |
| --- | --- | --- | --- | --- | --- | --- | --- | --- |
| 24 | Fu et al.(25) | Glucose Reduction & Nephroprotective | *P. sibiricum* | Indirect | STZ-induced diabetic SD rats | ↓ Fibrogenic factors: TGF-β1 (Transforming growth factor beta-1) ET-1 (Endothelin-1) Collagen I (Extracellular matrix deposition) | ↓ Blood glucose (P<0.05);  ↓ 24h proteinuria (P<0.05);  ↓ Serum creatinine/urea nitrogen; ↓ Renal TGF-β1, ET-1, Collagen I (IHC/WB, P<0.05); Improved renal histology (MASSON staining) | The protective effect of *polygonatum sibiricum* polysaccharide on diabetie nephropathy rats |
| 25 | Zhang(26) | Glucose Reduction &  Anti-fibroticn | *P. sibiricum* | Indirect | STZ-induced diabetic SD rats | ↑ BMP-7/Smad7 (Anti-fibrotic signals) ↓ TGF-β1/Smad2/3 (Pro-fibrotic signals) | ↓ FBG (24.81→13.28 mmol/L, P<0.05);  ↓ Heart mass index (4.16→3.21, P<0.05); ↓ Myocardial fibrosis (Masson staining); Normalized BMP-7/TGF-β1/Smad2/3/Smad7 (IHC, P<0.05) | Effect of *Polygonatum sibiricum* polysaccharides on myocardial fibrosis inrats with diabetes |
| 26 | Liu et al.(27) | Glucose Reduction & Nephroprotective | Unspecified species | Direct | STZ/HFD-induced diabetic nephropathy SD rats | ↓ Transferrin/FTH1 (Iron overload mitigation) ↑ GPX4 (Lipid peroxidation defense) ↓ MDA; ↑ GSH (Oxidative stress rescue) | ↓ Blood glucose/body weight (P<0.01); ↓ SCr/BUN/24h proteinuria (P<0.01); ↓ Renal iron/MDA; ↑ GSH (P<0.01); ↓ Transferrin/FTH1 mRNA & protein (P<0.01); ↑ GPX4 mRNA & protein (P<0.01); Improved histopathology  (HE: ↓ glomerulosclerosis) | Effects of Polygona-polysaccharose on Ferroptosis in Diabetic Nephropathy Rats |
| 27 | Chen et al.(28) | Glucose Reduction | *P. sibiricum* | Direct & Indirect | Genetic T2DM model (db/db mice) | ↑ Serum insulin/leptin ↑ Hepatic glycogen storage ↓ PEPCK1 (Gluconeogenesis suppression) Gut microbiota remodeling:  ↑ Beneficial genera; ↓ Harmful genera | ↓ Blood/urine glucose (P<0.05); ↑ Insulin/leptin (serum) (P<0.05); ↓ Hepatic PEPCK1 mRNA (P<0.01); Improved liver  histopathology & glycogen ↑; Microbiota shifts: Beneficial/harmful genus ratio ↑ (P<0.01); RDA: Significant flora-blood glucose/liver oxidative stress correlations | Hypoglycemic mechanisms of *Polygonatum sibiricum* polysaccharide in db/db mice via regulation of glycolysis/gluconeogenesis pathway and alteration of gut microbiota |
| 28 | Chen(29) | Glucose & Lipids Reduction | Unspecified species | Indirect | STZ/HFD-induced T2DM SD rats | ↓ TNF-α → Improved insulin signaling → ↓ Insulin resistance (HOMA-IR) | ↓ FPG (P<0.01); ↓ TC/TG/LDL-C (P<0.01); ↓ Atherogenic index (P<0.01); ↓ HOMA-IR (P<0.05); Improved hepatic histopathology | Effects of Polygonatum on Glycolipid Metabolism and TNF-α Levels in Type 2 Diabetic Rats |

**Table S1.** Cont

| **NO.** | **References** | **Key Effect** | **Species** | **Mechanism** | **Model System** | **Key Pathway/Targets** | **Outcome** | **Title** |
| --- | --- | --- | --- | --- | --- | --- | --- | --- |
| 29 | Zuo et al.(30) | Glucose Reduction & Antioxidant | *P.kingianum* | Indirect | STZ-induced diabetic SD rats | ↑ Nrf2/HO-1 mRNA | ↓ FBG & GHb (P<0.05);  Plasma: ↑ SOD/GSH; ↓ H₂O₂ (P<0.01);  ↑ T-AOC (aqueous-high);  ↑ SOD (aqueous-low);  ↓ MDA (P<0.01); ↑ Nrf2/HO-1 mRNA  (all treatment groups, P<0.001) | Effects of *Polygonatum Kingianum* Coll. et Hemsl on Oxidative Stress and Expression of Nrf2/HO-1 Signaling Pathyway in Diabetes Rats with Skin Lesions |
| 30 | Cao(31) | Glucose Reduction & Anti-Osteoporotic | *P. cyrtonema* | Direct | STZ-induced Zebrafish larvae | ↑ Osteogenic gene expression: Runx2b, Sp7, Col1a2 (Bone matrix formation) Sparc (Mineralization) Vdrb (Calcium homeostasis) | ↓ Tissue glucose (P<0.05); ↑ Skull mineralization area &  bone density (P<0.01); Gene upregulation: Runx2b/Sp7/Col1a2 (P<0.001); Sparc/Vdrb (P<0.01) | Pharmacodynamics study of polysaccharide from *Polygonatum cyrtonema* on zebrafish model with type 2 diabetic and osteoporosis |
| 31 | Zhang(32) | Glucose & Lipids Reduction | *P.kingianum* | Direct | HFD/STZ-induced T2DM ICR mice | Glycogen synthesis ↑ (liver/muscle) Glucose clearance ↑  (OGTT improvement) Lipid profile modulation (↓TC/TG/LDL; ↑HDL) | ↓ FBG (P<0.01); ↓TC/TG/LDL (P<0.01);  ↑HDL (P<0.05); Reduced islet damage | Antidiabetic Effect of Polysaccharides from *Polygonatum Kingianum* in Streptozotocin-induced Diabetic Mice |
| 32 | Miao et al.(33) | Glucose & Lipids Reduction | *P. sibiricum* | Direct | STZ/HFD-induced T2DM SD rats | NM | ↓ FBG (P<0.05 vs. model); ↓ TC/TG/LDL-C (P<0.05); ↑ HDL-C (P<0.05); Improved OGTT tolerance | Effects of PSP and PSP1 on Blood Glucose and Blood Lipid Levels in Type 2 Diabetes Mellitus Rats |
| 33 | Zeng et al.(34) | Lipids Reduction | *P. sibiricum* | Direct | HFD-induced obese/NAFLD rats | AMPK/SREBP-2/LDLR axis | ↓ Body weight (P<0.01); ↓ Serum TC/TG/LDL-C (28-32%); ↑ HDL-C (24%); ↓ Hepatic TNF-α/IL-1β/IL-6 (35-42%); ↑ SOD/CAT/GSH-Px (1.8-2.2x) | *Polygonatum sibiricum* polysaccharides protect against obesity and non-alcoholic fatty liver disease in rats fed a high-fat diet |
| 34 | Dong et al.(35) | Glucose & Lipids Reduction | *P.kingianum* | Indirect | HFD-induced dyslipidemic rats | miR-484-*Bacteroides****/****Roseburia* axis miRNA-microbiota crosstalk | ↓ miR-122/184/378b; ↑ 29 gut microbes (e.g. *Parabacteroides*) | Crosstalk Between *Polygonatum kingianum*, the miRNA, and Gut Microbiota in the Regulation of Lipid Metabolism |
| 35 | Su et al.(36) | Lipids & Hypertension Reduction | *P.kingianum* | Direct & Indirect | Metabolic Hypertensive Rats | Gut-Vascular Axis (LPS-TLR4/SCFAs-NO) | ↓ Systolic BP (25-32 mmHg); ↓ Serum LDL/TG (P<0.01); ↑ Gut barrier integrity (TEM confirmed); ↓ Serum LPS (63%) | The beneficial effects of *Polygonatum sibiricum* Red. superfine powder on metabolic hypertensive rats via gut-derived LPS/TLR4 pathway inhibition |

**Table S1.** Cont

| **NO.** | **References** | **Key Effect** | **Species** | **Mechanism** | **Model System** | **Key Pathway/Targets** | **Outcome** | **Title** |
| --- | --- | --- | --- | --- | --- | --- | --- | --- |
| 36 | Yang et al.(37) | Lipid Reduction | *P.kingianum* | Indirect | HFD-induced dyslipidemic rats | Multi-metabolic pathway regulation: Amino acid biosynthesis (Phe/Tyr/Trp/Val/Leu/Ile) Lipid metabolism (glycerophospholipid/arachidonic acid/sphingolipid) Carbohydrate metabolism (starch/sucrose) Vitamin cofactor metabolism (nicotinate/nicotinamide) | ↓ Serum/liver TC & TG (P<0.01);  Core pathways: Tryptophan metabolism; Linoleic acid metabolism | Integrated metabolomic profiling for analysis of antilipidemic effects of *Polygonatum kingianum* extract on dyslipidemia in rats |
| 37 | Liu et al.(38) | Lipid & Inflammation Reduction | *P. sibiricum* | Direct | HFD-obese mice | AMPK pathway activation ↓ SREBP-1/FAS (lipogenesis) ↑ PPARα/CPT-1 (fatty acid oxidation) Anti-inflammatory action: ↓ TNF-α/IL-6/IL-1β/iNOS | ↓ Body weight (P<0.01); ↓ Visceral fat (37%); ↓ Serum TG/TC (29-33%); ↑ Adiponectin (1.8x) & ↓ Resistin (52%) | *Polygonatum sibiricum* F. Delaroche polysaccharide ameliorates HFD‑induced mouse obesity via regulation of lipid metabolism and inflammatory response |
| 38 | Yang et al.(39) | Lipids & Atherosclerosis Reduction | *P. sibiricum* | Direct | In vivo:Atherosclerotic Rabbits In vitro: Endothelial Cells | Lipid metabolism regulation: ↓ TC/LDL-C/Lp(a) Endothelial protection: ↓ H2O2/LPS-induced apoptosis | ↓ Aortic foam cells (41-53%, P<0.01);  ↓ Serum TC/LDL-C/Lp(a) (P<0.01); ↑ Endothelial cell viability  (H2O2↑32%, LPS↑28%);  Dose-dependent efficacy  (3.2 ml/kg optimal) | Hypolipidemic Activity and Antiatherosclerotic Effect of Polysaccharide of *Polygonatum sibiricumin* Rabbit Model and Related Cellular Mechanisms |
| 39 | Wang(40) | Glucose & Lipids Reduction | *P.kingianum* | Indirect | HFD-induced SD rats | ↓*Firmicutes****/****Bacteroidetes* ratio  ↑*Roseburia****,*** *Bifidobacterium* ↑ZO-1/occludin expression  ↓LPS translocation→ ↓TNF-α/IL-1β & ↑IL-10 Activation of PPARγ signaling | ↓ Body weight, liver index, epididymal fat index; ↓ Serum TC, TG, LDL-C; ↑ HDL-C; ↓ FBG & serum insulin;  ↑ insulin sensitivity; Improved oral glucose tolerance ↑ Adiponectin; ↓ Leptin (LEP); Alleviated hepatic steatosis & adipocyte hypertrophy; ↓ Portal LPS & systemic inflammation | Effects and mechanisms of polysaccharide from *Polygonatum kingianum* on lipid metabolism disorder in rats |
| 40 | Liu et al.(41) | Glucose & Lipids Reduction & Hepatoprotective | *P. cyrtonema* | Direct & Indirect | HFD-induced C57BL/6J mice | Hepatic protection; Antioxidant; Microbiota | ↓ ALT, AST, hepatic steatosis;  ↓ TC, TG, LDL-C; ↑ HDL-C; ↑ SOD, GSH; ↓ MDA; ↑ *Allobaculum****,*** *Bifidobacterium*;  ↓ *Helicobacter*; Improved OGTT tolerance | Structural elucidation and anti-nonalcoholic fatty liver disease activity of *Polygonatum cyrtonema* Hua polysaccharide |

**Table S1.** Cont

| **NO.** | **References** | **Key Effect** | **Species** | **Mechanism** | **Model System** | **Key Pathway/Targets** | **Outcome** | **Title** |
| --- | --- | --- | --- | --- | --- | --- | --- | --- |
| 41 | Nie(42) | Glucose & Lipids Reduction | *P. sibiricum* | Direct | HFD/STZ-T2D mice | ABCA1 upregulation | ↓ FBG (P＜0.01); ↓ HOMA-IR;  ↓ TG/TC/LDL-C; ↑ HDL-C; ↑ ABCA1 mRNA/protein (P<0.01) | The influence of Polygonati Rhizoma on lipid metabolism disorder and ABCA1 gene expression in type 2 diabetic rats |
| 42 | Zhao et al.(43) | Glucose & Lipids Reduction | *P. sibiricum* | Direct | HFD/STZ-induced SD rats | ↓ SREBP-1c/SCD-1 → Hepatic lipogenesis ↓ | ↓ FBG, TG, TC, LDL-C, FFAs;  ↑HDL-C (P<0.05); ↓ Hepatic steatosis; ↓ SREBP-1c/SCD-1 protein (P<0.05) | Effects of Polygonatic Rhizome on the expression of SREBP-lc and SCD-l in type 2 diabetic rats |
| 43 | Kong et al.(44) | Lipids Reduction | *P. sibiricum* | Direct & Indirect | Yolk-emulsion-induced hyperlipidemic mice | Dual PPARα/β ↑ &  PPARγ/SREBP-1c ↓ ↓Lipogenesis ↑Fatty acid oxidation ↓Inflammation (TNF-α/IL-6) | ↓ Serum TC, TG, LDL-C;  ↑HDL-C (P<0.01); ↑ PPARα/β mRNA & protein (P<0.05); ↓ PPARγ/SREBP-1c mRNA  & protein (P<0.05); ↓ TNF-α/IL-6 expression (P<0.05) | Effect of polysaccharldes from  *Polygonatum sibiricum* on lipid-metabolism related mRNA and protein expression in hyperlipidemic mice |
| 44 | Yang et al.(18) | Lipids Reduction & Gut Microbiota Regulation | *P. sibiricum* | Indirect | In vitro: Simulated GI digestion Ex vivo: Fecal fermentation In vivo: T2DM mice gut microbiota analysis | ↓*Firmicutes****/****Proteobacteria*  ↑*Bacteroidetes* Modulates *Lactobacillus*, *Prevotella*, *Escherichia*, *Klebsiella* | Molecular weight reduction during digestion (24.19→20.39 kDa in stomach;  28.85→24.35 kDa in intestine); ↑Gut microbiota diversity & abundance | In *Vitro* Digestion Properties of *Polygonatum sibiricum* Polysaccharide and Its Regulatory Action on the Gut Microbiota in T2DM Mice |
| 45 | Wei(45) | Lipids Reduction | *P.kingianum* | Indirect | HFD-induced SD rats | Multi-tissue metabolic reprogramming: Amino acid metabolism  (Phe/Tyr/Trp biosynthesis) Lipid metabolism (Glycerophospholipids, Arachidonic/Linoleic acid); Cofactor metabolism (Riboflavin, Niacin) | ↓ Serum/hepatic TC/TG/LDL-C;  ↑HDL-C (P<0.05); 91 dysregulated metabolites across 4 matrices;  12 metabolic pathways normalized (Phe/Tyr/Trp, AA biosynthesis, lipid/cofactor metabolism) | Effects and mechanisms of *Polygonatum kingianum* on lipid metabolism disorder in rats based on metabolomics |
| 46 | Guo et al.(46) | Lipids Reduction & Anti-Atherosclerotic | *P. cyrtonema* | Indirect | HFD-induced atherosclerosis in  LDLr⁻/⁻ mice | ↓ NF-κB: p-p65↓ + IκBα↑ ↓ MAPKs: p-p38↓ + p-ERK1/2↓ ↓ Akt phosphorylation; | ↓ Atherosclerotic lesions  (P<0.01; males > females);  ↓ Oxidative damage/inflammation (P<0.05); TC/TG/LDL-C↓ | The interventional effect of *Polygonatum cyrtonema* Hua polysaccharide on  atherosclerosis in mice of different sexes |

| **NO.** | **References** | **Key Effect** | **Species** | **Mechanism** | **Model System** | **Key Pathway/Targets** | **Outcome** | **Title** |
| --- | --- | --- | --- | --- | --- | --- | --- | --- |
| 47 | Bao(47) | Lipids Reduction & Microbiome-Modulating | *P. sibiricum* | Indirect | HFD-induced obese mice | ↓ Hepatic inflammation (TNF-α/IL-6) ↑ Beneficial microbiota (*Lactobacillus*↑) ↓ Pathogenic bacteria Lipid metabolism modulation | Metabolic improvements:  ↓ Body weight (-16.13%); ↓ Fat index (-18.79%);  ↓ Liver index (-15.70%); ↓ TC (-43.19%), TG (-23.07%),  LDL-C (-10.90%) Anti-inflammatory:  ↓ TNF-α (-26.48%), ↓ IL-6 (-37.70%) Microbiome remodeling:  ↑ *Firmicutes/Bacteroidetes*  (91.94% total) ↑ *Lactobacillus* (+20.51% vs. HFD) ↓ Pathogenic genera | Preparation of *Polygonatum sibiricum* Polysaccharide by Microbial Method and Its Lipid-lowering Function |
| 48 | Zhu et al.(48) | Lipids Reduction & Antiatherosclerotic | Unspecified species | Direct | Atherogenic diet-induced hamster model | Serum lipid profile normalization Apolipoprotein regulation Endothelial dysfunction amelioration | ↓ TC, LDL-C (p<0.01); ↓ Atherosclerotic lesions;  ↓ Oxidative stress: ↓ MDA, ↑ SOD; ↓ Inflammation: ↓ TNF-α, TXA2; | Antiatherosclerotic Potential of Rhizoma Polygonati Polysaccharide in Hyperlipidemia-induced Atherosclerotic Hamsters |
| 49 | Wang et al.(49) | Glucose Reduction & Retinal protection | *P. sibiricum* | Indirect | STZ-induced diabetic SD rats | Apoptosis regulation;  Growth factor signaling | ↑Bcl-2, ↓Bax; ↓EGF, ↓VEGF, ↓TGF-β, ↓p38 MAPK Reduced vascular leakage, tortuosity, and apoptosis in retinal ganglion cells. | *Polygonatum sibiricum* polysaccharide potentially attenuates diabetic retinal injury in a diabetic rat model |
| 50 | Gong et al.(50) | Lipids Reduction & Gut Microbiota Regulation | *P. cyrtonema* | Indirect | DSS-induced Colitis C57BL/6 mice | Anti-inflammatory; Gut barrier repair; Microbiota regulation | ↓ IL-6, iNOS, COX-2; ↑ Occludin, ZO-1; ↑ *Muribaculaceae*, *Lactobacillus*;  ↓ *Bacteroides*, *Escherichia-Shigella* | Structural characteristics of steamed Polygonatum cyrtonema polysaccharide and its bioactivity on colitis via improving the intestinal barrier and modifying the gut microbiota |

The following are the references mentioned in the table:

1. Li R, Tao A, Yang R, Fan M, Zhang X, Du Z, et al. Structural characterization, hypoglycemic effects and antidiabetic mechanism of a novel polysaccharides from Polygonatum kingianum Coll. et Hemsl. Biomed Pharmacother. 2020;131:110687.

2. Gu W, Wang Y, Zeng L, Dong J, Bi Q, Yang X, et al. Polysaccharides from Polygonatum kingianum improve glucose and lipid metabolism in rats fed a high fat diet. Biomed Pharmacother. 2020;125:109910.

3. Cai J, Zhu Y, Zuo Y, Tong Q, Zhang Z, Yang L, et al. Polygonatum sibiricum polysaccharide alleviates inflammatory cytokines and promotes glucose uptake in high‑glucose‑ and high‑insulin‑induced 3T3‑L1 adipocytes by promoting Nrf2 expression. Molecular Medicine Reports. 2019.

4. Cai J-L, Li X-P, Zhu Y-L, Yi G-Q, Wang W, Chen X-Y, et al. Polygonatum sibiricum polysaccharides (PSP) improve the palmitic acid (PA)-induced inhibition of survival, inflammation, and glucose uptake in skeletal muscle cells. Bioengineered. 2021;12(2):10147-59.

5. Wang G, Liu Z, Liang D, Yu J, Wang T, Zhou F, et al. Aqueous extract of Polygonatum sibiricum ameliorates glucose and lipid metabolism via PI3K/AKT signaling pathway in high‐fat diet and streptozotocin‐induced diabetic mice. Journal of Food Biochemistry. 2022;46(12).

6. Yan H, Lu J, Wang Y, Gu W, Yang X, Yu J. Intake of total saponins and polysaccharides from Polygonatum kingianum affects the gut microbiota in diabetic rats. Phytomedicine. 2017;26:45-54.

7. Zhang Y. The therapeutic effect of polygonatum sibiricum polysaccharid on DN rats and the possible mechanism [硕士]2011.

8. Jia L, Shi J, Duan Z, Dong R, Wang M, Zheng J. Effects of Polygonati Rhizoma Polysaccharide on glucose metabolism in diabetic mice induced by high fat diet. China Medical Herald. 2017;14(08):24-8.

9. Wang G, Li J, He L, Li Y, Wang J, Jia S, et al. Study on the prevention of glyeolipid metabolism disorder in mice by polygonatum sibiricum polysaccharides. Journal of Food Safety & Quality. 2020;11(21):7829-36.

10. Xie S-Z, Zhang W-J, Liu W, Bai J-B, Xie S-L, Wang T, et al. Physicochemical characterization and hypoglycemic potential of a novel polysaccharide from Polygonatum sibiricum Red through PI3K/Akt mediated signaling pathway. Journal of Functional Foods. 2022;93.

11. Zhang H, Li H, Pan B, Zhang S, Su X, Sun W, et al. Integrated 16S rRNA Sequencing and Untargeted Metabolomics Analysis to Reveal the Protective Mechanisms of *Polygonatum sibiricum* Polysaccharide on Type 2 Diabetes Mellitus Model Rats. Current Drug Metabolism. 2023;24(4):270-82.

12. Teng H, Zhang Y, Jin C, Wang T, Huang S, Li L, et al. Polysaccharides from steam‐processed Polygonatum cyrtonema Hua protect against d‐galactose‐induced oxidative damage in mice by activation of Nrf2/HO‐1 signaling. Journal of the Science of Food and Agriculture. 2022;103(2):779-91.

13. Yang G. The Effect of Polygonatum Cyrtonema Hua Polysaccharides（PCP） on the Expression and Secretion of GLP-1 and its Molecular Mechanism [Master]: Hefei University of Technology; 2018.

14. Gao Y, Ye X, Li X, Zhu X, Wang L, Huang W, et al. Extract of Polygonatum sibiricum and their inhibition of α-glucosidase. Chinese Traditional Patent Medicine. 2010;32(12):2133-7.

15. Wang Y, Qin S, Pen G, Chen D, Han C, Miao C, et al. Original Research: Potential ocular protection and dynamic observation of Polygonatum sibiricum polysaccharide against streptozocin-induced diabetic rats' model. Exp Biol Med (Maywood). 2017;242(1):92-101.

16. Zeng L, Xiang R, Zhang Y, Fu C, Li G, Li C. Hypoglycemic effect and mechanism of Polygonatum sibiricum polysaccharides on diabetic mice. Chinese Traditional Patent Medicine. 2022;44(09):2989-94.

17. Ren Q, Zhang X, Wang M, Li x, Yao Y, Ran Y, et al. Study on hypoglyeemie effeet and intestinal effeet of Polygonatum sibiricum polysaccharides in diabetic mice. Journal of Pharmaceutical Practice and Service. 2022;40(6):510-4.

18. Yang M, Yuan M, Lu W, Bao Y, Chai Y. In Vitro Digestion Properties of Polygonatum sibiricum Polysaccharide and Its Regulatory Action on the Gut Microbiota in T2DM Mice. Modern Food Science ＆ Technology. 2021;37(08):14-21.

19. Wang G. The Protective Effects of Polygonatum Sibricum Aqueous Extract on Glycolipid metabolism in Mice Based on PI3K/AKT Pathway [master]: Anhui Medical University; 2021.

20. Dong M. To explore the mechanism of Polygonum polygonum polysaccharide in alleviating T2DM based on mitophagy mediated by PINK1/Parkin [Master]2023.

21. Liu Z, Xu J, Liang Z, Yang H, Chen Y. Effect of Polygonatum Yunnanensis Polysaccharide on Diabetic Nephropathy Mice Caused by Low Concentration of Streptozotocin. 中医药导报. 2021;27(1):12-5.

22. Sun T. Based on the theory of "Yin deficiency is often insufficient", a study on the improvement of reproductive damage in diabetic male rats by Polygonati Rhizoma through the AMPK-mTOR-ULK1 pathway was explored [Master]2024.

23. Dong Q, Dong K, Zhang C. Effects of rhizoma polygonati on the expression of glucose transporter-4 gene in type 2 diabetes mellitus rats with insulin resistance. Journal of Xinxiang Medical University. 2012;29(07):493-5.

24. Chen X, Lai Y, Wang C. Experimental Investigation of *Polygonatum kingianum* on Blood Glucose in Induced Hyperglycemic Mice. Lishizhen Medicine and Materia Medica Research. 2010;21(12):3163-4.

25. Fu T, Wang G, Chen T, Zhang Z. The protective effect of *polygonatum sibiricum* polysaccharide on diabetie nephropathy rats. Pharmacology and Clinics of Chinese Materia Medica. 2015;31(04):123-6.

26. Zhang Z, Wang G, Chen T, Fu T. Effect of Polygonatum sibiricum polysaccharides on myocardial fibrosis inrats with diabetes. Chinese Journal of Public Health. 2016;32(06):807-10.

27. Liu H, LI H, Zhang H, Lu S, Su X, Pan H. Effects of Polygona-polysaccharose on Ferroptosis in Diabetic Nephropathy Rats. Chinese Journal of Information on Traditional Chinese Medicine. 2023;30(08):126-30.

28. Chen X, Tong YL, Ren ZM, Chen SS, Mei XY, Zhou QY, et al. Hypoglycemic mechanisms of Polygonatum sibiricum polysaccharide in db/db mice via regulation of glycolysis/gluconeogenesis pathway and alteration of gut microbiota. Heliyon. 2023;9(4):e15484.

29. Chen Y. Effects of Polygonatum on Glucose and Lipid Metabolism and TNF-α Levels in Type 2 Diabetic Rats [Master]: Beijing University of Chinese Medicine; 2018.

30. Zuo X, Xu Y, Qiu B, Li J, Yu J. Effects of *Polygonatum Kingianum* Coll. et Hemsl on Oxidative Stress and Expression of Nrf2/HO-1 Signaling Pathyway in Diabetes Rats with Skin Lesions. World Science and Technology-Modernization of Traditional Chinese Medicine. 2023;25(09):2959-66.

31. Cao Y, Wang K, Wang Z, BIian J, An L, Xu S, et al. Pharmacodynamics study of polysaccharide from *Polygonatum cyrtonema* on zebrafish model with type 2 diabetic and osteoporosis. Chinese Traditional and Herbal Drugs. 2021;52(21):6545-51.

32. Zhang X, Duan B, Tao A, GUO W, Meng G, Yang S. Antidiabetic Effect of Polysaccharides from Polygonatum Kingianum in Streptozotocin-induced Diabetic Mice. Chinese Journal of Ethnomedicine and Ethnopharmacy. 2022;31(12):19-24.

33. Miao X, Luo P, Hu S, Zheng M, Liu C. Effects of PSP and PSP1 on Blood Glucose and Blood

Lipid Levels in Type 2 Diabetes Mellitus Rats. Journal of Hubei University of Science and Technology. 2022;36(02):101-4+17.

34. Zeng L, Zhong F, Chen Z, Li G, Zhu Q. Polygonatum sibiricum polysaccharides protect against obesity and non-alcoholic fatty liver disease in rats fed a high-fat diet. Food Science and Human Wellness. 2022;11(4):1045-52.

35. Dong J, Gu W, Yang X, Zeng L, Wang X, Mu J, et al. Crosstalk Between Polygonatum kingianum, the miRNA, and Gut Microbiota in the Regulation of Lipid Metabolism. Frontiers in Pharmacology. 2021;12.

36. Su J, Wang Y, Yan M, He Z, Zhou Y, Xu J, et al. The beneficial effects of Polygonatum sibiricum Red. superfine powder on metabolic hypertensive rats via gut-derived LPS/TLR4 pathway inhibition. Phytomedicine. 2022;106.

37. Yang XX, Wei JD, Mu JK, Liu X, Dong JC, Zeng LX, et al. Integrated metabolomic profiling for analysis of antilipidemic effects of *Polygonatum kingianum* extract on dyslipidemia in rats. World J Gastroenterol. 2018;24(48):5505-24.

38. Liu B, Tang Y, Song Z, Ge J. Polygonatum sibiricum F. Delaroche polysaccharide ameliorates HFD‑induced mouse obesity via regulation of lipid metabolism and inflammatory response. Mol Med Rep. 2021;24(1).

39. Yang J-x, Wu S, Huang X-l, Hu X-q, Zhang Y. Hypolipidemic Activity and Antiatherosclerotic Effect of Polysaccharide of *Polygonatum sibiricumin* Rabbit Model and Related Cellular Mechanisms. Evidence-Based Complementary and Alternative Medicine. 2015;2015:1-6.

40. Wang Y. Effects and mechanisms of polysaccharide from Polygonatum kingianum on lipid metabolism disorder in rats [master]2017.

41. Liu W, Shao T, Tian L, Ren Z, Gao L, Tang Z, et al. Structural elucidation and anti-nonalcoholic fatty liver disease activity of Polygonatum cyrtonema Hua polysaccharide. Food & Function. 2022;13(24):12883-95.

42. Nie Y. The influence of Polygonati Rhizoma on lipid metabolism disorder and ABCA1 gene expression in type 2 diabetic rats [硕士]: Jiamusi University; 2006.

43. Zhao H, Xu Y, Zhao H, Liu Y, Zhao W. Effects of Polygonatic Rhizome on the expression of SREBP-lc and SCD-l in type 2 diabetic rats. Pharmacology and Clinics of Chinese Materia Medica. 2015;31(1):106-9.

44. Kong X, Liu J, Li H, Chen Z. Effect of polysaccharldes from

*Polygonatum sibiricum* on lipid-metabolism related mRNA and protein expression in hyperlipidemic mice. CHINA JOURNAL OF CHINESE MATERIA MEDICA. 2018;43(18):3740-7.

45. Wei J. Effects and mechanisms of *Polygonatum kingianum* on lipid metabolism disorder in rats based on metabolomics [硕士]2018.

46. Guo A, Li X, Pan L, Li Q, Luo J, Zha X. The interventional effect of Polygonatum cyrtonema Hua polysaccharide on atherosclerosis in mice of different sexes. Food Science and Human Wellness. 2024;13(1):370-80.

47. Bao Z. Preparation of Polygonatum sibiricum Polysaccharide by Microbial Method and Its Lipid-lowering Function [master]2021.

48. Zhu X, Li Q, Lu F, Wang H, Yan S, Wang Q, et al. Antiatherosclerotic Potential of Rhizoma Polygonati Polysaccharide in Hyperlipidemia-induced Atherosclerotic Hamsters. Drug Res (Stuttg). 2015;65(9):479-83.

49. Wang Y, Lan C, Liao X, Chen D, Song W, Zhang Q. Polygonatum sibiricum polysaccharide potentially attenuates diabetic retinal injury in a diabetic rat model. J Diabetes Investig. 2019;10(4):915-24.

50. Gong H, Gan X, Qin B, Chen J, Zhao Y, Qiu B, et al. Structural characteristics of steamed Polygonatum cyrtonema polysaccharide and its bioactivity on colitis via improving the intestinal barrier and modifying the gut microbiota. Carbohydrate Polymers. 2024;327.
